# Prognostic factors associated with primary non-responsiveness to antibiotics and appendicitis recurrence for CT-diagnosed uncomplicated acute appendicitis: secondary analysis of two randomized clinical trials

**DOI:** 10.1093/bjs/znaf143

**Published:** 2025-07-31

**Authors:** Liisa Selänne, Saija Hurme, Suvi Sippola, Tero Rautio, Pia Nordström, Tuomo Rantanen, Tarja Pinta, Imre Ilves, Anne Mattila, Eeva-Liisa Sävelä, Jukka Rintala, Hannu Paajanen, Juha Grönroos, Jussi Haijanen, Paulina Salminen

**Affiliations:** Division of Digestive Surgery and Urology, Turku University Hospital, Turku, Finland; Department of Surgery, University of Turku, Turku, Finland; Department of Biostatistics, University of Turku and Turku University Hospital, Turku, Finland; Department of Surgery, Helsinki University Hospital, Helsinki, Finland; Department of Surgery, Oulu University Hospital, Oulu, Finland; Medical Research Centre Oulu, University of Oulu, Oulu, Finland; Department of Gastroenterology and Alimentary Tract Surgery, Tampere University Hospital, Tampere, Finland; Faculty of Medicine and Health Technology, University of Tampere, Tampere, Finland; Department of Surgery, Kuopio University Hospital, Kuopio, Finland; Department of Surgery, Institute of Clinical Medicine, University of Eastern Finland, Kuopio, Finland; Department of Surgery, Seinäjoki Central Hospital, Seinäjoki, Finland; Department of Surgery, Mikkeli Central Hospital, Mikkeli, Finland; Department of Surgery, Wellbeing Services County of Central Finland/Hospital Nova of Central Finland, Jyväskylä, Finland; Department of Surgery, Satakunta Central Hospital, Pori, Finland; Department of Surgery, Oulu University Hospital, Oulu, Finland; Department of Surgery, Rovaniemi Central Hospital, Rovaniemi, Finland; Department of Surgery, Mikkeli Central Hospital, Mikkeli, Finland; Division of Digestive Surgery and Urology, Turku University Hospital, Turku, Finland; Department of Surgery, University of Turku, Turku, Finland; Division of Digestive Surgery and Urology, Turku University Hospital, Turku, Finland; Department of Surgery, University of Turku, Turku, Finland; Division of Digestive Surgery and Urology, Turku University Hospital, Turku, Finland; Department of Surgery, University of Turku, Turku, Finland

## Abstract

**Background:**

Antibiotics are safe and efficient for CT-diagnosed uncomplicated acute appendicitis. Identifying predictive factors of primary non-responsiveness or recurrence would further improve antibiotic treatment success and safety.

**Methods:**

All patients treated with antibiotics in two large RCTs (APPAC and APPAC II) were included. The primary non-responsiveness analysis compared patients operated on within 30 days after randomization for complicated appendicitis with either patients presenting with uncomplicated appendicitis at surgery within 30 days or patients with successful antibiotic treatment during 3-year follow-up. Prognostic factors for appendicitis recurrence were assessed by comparing patients with successful antibiotic treatment with patients with acute appendicitis operated on after 30 days of initial antibiotics.

**Results:**

Of 856 patients randomized to antibiotics (mean(s.d.) age of 36(12) years; 365 (42.6%) were women), 832 were eligible for non-responsiveness analysis and 732 for appendicitis recurrence analysis. Findings associated with primary non-responsiveness on admission included an appendiceal diameter ≥15 mm (adjusted risk ratio (RR) 4.00 (95% c.i. 2.00 to 7.92) (*P* < 0.001)) and a body temperature >38°C (adjusted RR 2.76 (95% c.i. 1.27 to 6.03) (*P* = 0.011)). During the first 6–30 h after admission, C-reactive protein (CRP) ≥100 mg/l (negative predictive value of 99%) and leucocyte count ≥9 × 10^9^/l were associated with primary non-responsiveness (adjusted RR 8.29 (95% c.i. 3.69 to 18.63) (*P* < 0.001) and adjusted RR 4.44 (95% c.i. 1.79 to 11.05) (*P* = 0.001) respectively). No prognostic findings for appendicitis recurrence were identified.

**Conclusion:**

Patients with an appendiceal diameter ≥15 mm and a body temperature >38°C may not be optimal candidates for non-operative treatment for uncomplicated acute appendicitis. Patients with CRP <100 mg/l at 24 h of antibiotic treatment for uncomplicated acute appendicitis have a 99% likelihood of successful antibiotic therapy.

**Registration numbers:**

NCT03236961 and NCT01022567 (http://www.clinicaltrials.gov).

## Teaser

The aim of this study was to assess possible preinterventional on-admission findings and post-treatment findings during the first day that could be associated with primary non-responsiveness to antibiotics and to evaluate possible baseline prognostic factors for appendicitis recurrence after initial successful antibiotic treatment of uncomplicated acute appendicitis. In the analysis assessing primary non-responsiveness to initial antibiotics, patients with an appendiceal diameter ≥15 mm or a body temperature >38°C on admission were found to have an increased risk of primary antibiotic non-responsiveness. In addition, the immediate postintervention findings of C-reactive protein ≥100 mg/l and leucocyte count ≥9 × 10^9^/l during the first 6–30 h after admission were also found to be associated with primary antibiotic non-responsiveness. There were no prognostic factors for appendicitis recurrence.

## Introduction

According to current understanding, appendicitis can be divided by severity into two different disease entities, uncomplicated and complicated acute appendicitis^1–3^, enabling different treatments. The long-standing belief that all instances of appendicitis invariably lead to perforation unless patients are operated on^4,5^ appears to be applicable only to complicated acute appendicitis and the majority of instances of appendicitis are uncomplicated. Contemporary studies show that uncomplicated acute appendicitis can be safely and efficiently managed with antibiotics^6–10^ and also in an outpatient setting^8,11,12^. However, there is still a lack of established and consistent criteria for distinguishing between uncomplicated and complicated acute appendicitis. Consequently, the objective findings minimizing non-responsiveness to initial antibiotics and therefore facilitating safe outpatient management for patients with uncomplicated appendicitis are yet to be defined.

The presence of an appendicolith has been shown to be associated with an increased risk of primary non-responsiveness to antibiotics^10,13–15^ and is therefore often considered as a feature of complicated appendicitis. Furthermore, in a secondary analysis of the APPAC II trial assessing the potential preintervention findings associated with primary non-responsiveness to antibiotics, an appendiceal diameter ≥15 mm and a body temperature >38°C were associated with non-responsiveness to antibiotics^16^ and added as further exclusion criteria in the ongoing APPAC IV double-blind RCT, comparing oral moxifloxacin and placebo for uncomplicated acute appendicitis in an outpatient setting^17^. Besides successful initial treatment, the long-term risk of possible recurrent appendicitis is a key component of optimizing non-operative management and patient satisfaction^18^. The longest follow-up to date is from the APPAC trial, reporting an appendectomy rate of 39.1% at 5 years after initial antibiotics based on the study protocol of appendectomy for all patients with suspected appendicitis recurrence. These results were recently corroborated by consistent 3-year findings of the APPAC II trial, with appendectomy rates of 36.6% after oral antibiotics only and 34.8% after intravenous followed by oral antibiotics. No preintervention prognostic factors for recurrent appendicitis have yet been identified.

The aim of this study was to assess possible preinterventional on-admission findings and post-treatment findings during the first day that could be associated with primary non-responsiveness to antibiotics, adding to the previously reported secondary analysis of the APPAC II trial^16^ by combining patients from the APPAC trial and the APPAC II trial to achieve a larger sample size. The second novel aim was to evaluate possible baseline prognostic factors for appendicitis recurrence after initial successful antibiotic treatment of uncomplicated acute appendicitis. The primary objective was to further optimize patient selection and consequently increase the safety and efficacy of non-operative management of uncomplicated acute appendicitis.

## Methods

### Trial design, participants, and interventions

The patient population for this study was a combination of two earlier RCTs (APPAC and APPAC II^7,9,19,20^) assessing non-operative management of CT-confirmed uncomplicated acute appendicitis in patients aged 18–60 years. Briefly, the APPAC trial was a multicentre, open-label, non-inferiority RCT involving 530 patients at six hospitals in Finland^7,19^ and the APPAC II trial was a multicentre, open-label, non-inferiority RCT involving 603 patients at nine hospitals in Finland^9,20^. The trial protocols were approved by the ethics committee at the Hospital District of Southwest Finland and by institutional research boards at each participating site, the trials were performed in accordance with the Declaration of Helsinki, and all patients gave written informed consent. Criteria for uncomplicated acute appendicitis on CT included an appendiceal diameter >6 mm, a thickened, contrast-enhanced appendiceal wall, periappendiceal oedema and/or minor fluid collection, and the absence of criteria for complicated acute appendicitis (presence of an appendicolith, perforation, abscess, or suspicion of a tumour). Exclusion criteria included age <18 years or >60 years, peritonitis, contraindication for CT for example allergy to contrast media or iodine, pregnancy or lactation, kidney insufficiency or serum creatinine value exceeding the upper reference limit, metformin use, serious systemic illness (for example malignancy), failure to cooperate or give consent, and complicated acute appendicitis on CT. In the APPAC trial, all patients were randomized to either appendectomy or antibiotic treatment (intravenous ertapenem 1 g/day for 3 days followed by oral levofloxacin 500 mg/day and metronidazole 500 mg 3 times/day for 7 days)^19^. In the APPAC II trial, patients were randomized to receive either oral moxifloxacin (400 mg/day) for 7 days or intravenous ertapenem sodium (1 g/day) for 2 days followed by oral levofloxacin (500 mg/day) and metronidazole (500 mg 3 times/day) for 5 days^20^.

### Patients evaluated for factors associated with primary non-responsiveness to antibiotics

**Fig. 1:**
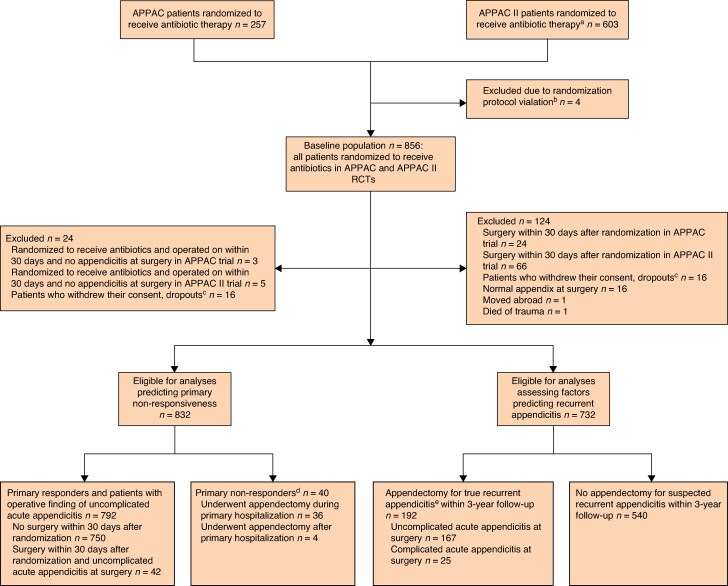
Flow of patients in a secondary analysis of two RCTs assessing prognostic factors associated with primary non-responsiveness to antibiotics and appendicitis recurrence for CT-diagnosed uncomplicated acute appendicitis ^a^Three hundred and two patients from the group that received oral antibiotics and 301 patients from the group that received intravenous antibiotics followed by oral antibiotics. ^b^Patients erroneously randomized, despite a finding of complicated acute appendicitis initially seen on CT imaging, were excluded from the analyses, according to the study protocol. ^c^Patients who withdrew consent within 24 h of randomization who received a maximum of one dose of the randomized treatment were excluded from the analyses. Two patients, who withdrew their consent 5 and 7 days after randomization, were included in the analyses. ^d^Surgery within 30 days after randomization and complicated acute appendicitis at surgery. ^e^Acute appendicitis confirmed by histopathological findings after appendectomy for suspected recurrence.

For analysis evaluating primary non-responsiveness to antibiotics within the first 3 years, all patients randomized to receive antibiotics within the APPAC trial (257 patients) and the APPAC II trial (603 patients) were assessed (*Fig. 1*). The patients undergoing appendectomy during the primary hospitalization with a finding of complicated acute appendicitis (40 patients) and either patients undergoing surgery for uncomplicated acute appendicitis within 30 days after randomization (42 patients) or patients with successful initial antibiotic treatment (no appendectomy within 30 days) (750 patients) were included in the analyses for the non-responsiveness. The patients undergoing surgery for uncomplicated acute appendicitis within the first 30 days were considered as having successful treatment, as the initial radiological diagnosis for the uncomplicated acute appendicitis was accurate at surgery. There were no official standardized criteria to proceed to appendectomy; this was left to the clinical judgement of the surgeon on call. To validate the accuracy of the final diagnosis in differentiating between uncomplicated and complicated acute appendicitis, two investigators (J.H. and S.S. or L.S.), who were unaware of each other’s evaluation, assessed the surgical and histopathological findings in a blinded manner. If there was disagreement, another investigator (P.S.) reviewed the diagnosis. The patients randomized to receive antibiotics and operated on within 30 days of randomization and with an operative finding of a normal appendix without inflammation were excluded, given that the initial diagnosis of appendicitis was incorrect (8 patients).

### Patients evaluated for factors predicting appendicitis recurrence

For analysis assessing possible prognostic factors predicting recurrent appendicitis within the first 3 years after initial successful antibiotic treatment, all patients treated with antibiotics within the APPAC trial (257 patients) and the APPAC II trial (603 patients) were assessed. The patients operated on within 30 days after randomization, that is the potential non-responders, were excluded, as the symptoms leading to an early operation were considered more likely to be caused by the initial acute appendicitis, rather than recurrent acute appendicitis (90 patients). Patients operated on after 30 days with a normal appendix according to histopathology were deemed as not having recurrent appendicitis and were also excluded.

True appendicitis recurrence was defined as histopathologically confirmed acute appendicitis after an appendectomy performed at least 30 days after initial antibiotic treatment. According to the study protocols of both RCTs^19,20^, there were no predefined diagnostic criteria for diagnosing recurrent appendicitis and the diagnosis was made clinically by the surgeon on call without repeated CT and laparoscopic appendectomy was performed for suspected recurrent appendicitis.

Patient outcomes were evaluated by telephone interviews conducted at 3 years after the diagnosis. Patients were asked about appendicitis recurrence and potential symptoms. For patients who were not reached for follow-up by telephone or clinical visit, a search for electronical hospital records was conducted in each hospital district to assess possible appendicitis recurrence.

### Outcome measures

The potential factors associated with antibiotic non-responsiveness registered on admission included age, sex, visual analogue scale (VAS) pain score, leucocyte count, body temperature, C-reactive protein (CRP), symptom duration, and appendiceal diameter on CT. CRP and leucocyte count were also analysed during the first 6–30 h after admission. This time frame was selected considering that the patients’ first follow-up typically occurs the morning after admission. Given that patients are admitted at varying times, the range was designed to accommodate these differences.

In the analysis assessing possible prognostic factors predicting appendicitis recurrence, preinterventional factors of patients with successful antibiotic treatment and patients with a true recurrence were compared. The evaluated potential prognostic factors on admission included age, sex, VAS score at emergency, leucocyte count, body temperature, CRP, and appendiceal diameter.

### Statistical analysis

Continuous variables are presented as mean(s.d.) or median (interquartile range) and differences between treatment groups were tested using one-way ANOVA or the Kruskal–Wallis test, depending on the distribution. Categorical variables are presented as *n* (%) and differences between groups were tested using the chi-squared test. Differences in baseline characteristics between different groups were tested to assure the similarity of patients in the APPAC and APPAC II studies.

Factors associated with antibiotic non-responsiveness (appendiceal diameter on CT imaging, CRP, leucocyte count, body temperature, age, sex, VAS score, and symptom duration) were analysed using a binomial generalized linear model (log-binomial model). Study centre was used in the model as a study design factor (random effect). Randomization group (3 categories) was also used as a study design factor (fixed effect) to account for the effects of study (APPAC and APPAC II) and different antibiotic treatments. Factors were evaluated first using a univariable approach and then significant variables were included in a multivariable model. Associations of the prognostic factors with outcomes are described using risk ratios (RRs) with 95% confidence intervals. Receiver operating characteristic (ROC) curves were used to assess the clinical value of CRP and leucocyte count on admission and 6–30 h after admission, and the difference between those values. Optimal cut-off points were evaluated for CRP and leucocyte count using clinical evaluation and ROC curves. Areas under the ROC curves (AUCs) with 95% confidence intervals are presented, as well as sensitivities, specificities, and negative predictive values, with 95% confidence intervals. A Cox proportional hazards model was used to evaluate the prognostic factors for recurrent appendicitis. Randomization group (3 categories) was used as a study design factor (fixed effect) in the model to account for the effects of study (APPAC and APPAC II) and different antibiotic treatments. Each prognostic factor was analysed separately to find significant factors, but, because there were no significant findings, no further analyses were performed. A proportional hazards assumption was evaluated using scaled Schoenfeld residuals, which were plotted against time for each variable to evaluate possible systematic trends. HRs with 95% confidence intervals are presented to describe the associations.

The analyses were based on the intention-to-treat principle. *P* < 0.050 was considered statistically significant. Statistical analyses were carried out using the SAS system for Windows^®^, version 9.4 (SAS Institute Inc., Cary, NC, USA) and figures were drawn using R 4.2.1 (R Foundation for Statistical Computing, Vienna, Austria).

## Results

See *Fig. 1* for the study flow chart. A total of 4891 patients (1379 in the APPAC trial and 3512 in the APPAC II trial) were assessed and 1133 patients (530 in the APPAC trial and 603 in the APPAC II trial) underwent randomization. Altogether, 856 patients (257 in the APPAC trial and 599 in the APPAC II trial) were randomized to receive antibiotics (mean(s.d.) age of 36(12) years; 365 (42.6%) were women). The baseline characteristics of the trial patients are presented in *Table 1*. The only statistically significant difference in the baseline demographics between these trials was appendiceal diameter on CT. The mean(s.d.) appendiceal diameter was 11.4(2.52) mm in the APPAC trial, 10.7(2.35) mm in the APPAC II trial for the group that received intravenous antibiotics followed by oral antibiotics, and 10.9(2.61) mm in the APPAC II trial for the group that only received oral antibiotics (*P* = 0.008). At 3-year follow-up of appendicitis recurrence, the combined follow-up rate was 99.6% (853 of 856).

**Table 1:** Patient baseline characteristics in the APPAC trial and the APPAC II trial

	All patients randomized to antibiotics (*n* = 856)	APPAC (intravenous + oral) (*n* = 257)	APPAC II (intravenous + oral) (*n* = 298)	APPAC II (oral) (*n* = 301)	*P*
Male sex	491 (57.4)	155 (60.3)	172 (54.5)	164 (57.7)	0.378§
Age (years), mean(s.d.)	36(12)	36(12)	35(11)	36(12)	0.552¶
VAS score for pain on admission, mean(s.d.)*	5.23(2.3)	5.23(2.3)	5.28(2.4)	5.20(2.3)	0.894¶
Body temperature (°C), mean(s.d.)	37.2(0.6)	37.2(0.6)	37.2(0.6)	37.2(0.6)	0.417¶
Leucocyte count (×10^9^/l), median (interquartile range)†	12.0 (9.1–14.8)	11.5 (8.9–14.2)	12.2 (9.1–14.9)	12.5 (9.4–14.6)	0.073#
CRP (mg/l), median (interquartile range)†	32.0 (11.0–62.0)	39.3 (11.0–64.0)	34.0 (12.0–61.0)	29.0 (11.0–60.0)	0.707#
Appendiceal diameter on CT imaging (mm), mean(s.d.)‡	11.0(2.51)	11.4(2.52)	10.7(2.35)	10.9(2.61)	0.008¶

Values are *n* (%) unless otherwise indicated. *Score range = zero to ten (a score of 0 indicates no pain and a score of 10 indicates the worst possible pain). †Reference range for leucocyte count = 3.4–8.2 × 10^9^/l and the reference value for CRP is <10 mg/l. ‡Defined as the outer to outer surface appendiceal diameter measured from the widest part of the appendix on the axial plane (that is perpendicular to the longitudinal axis). A diameter of ≤6 mm was considered normal, whereas a diameter >6 mm with signs of acute inflammation (thickened and contrast-enhancing appendiceal wall with periappendiceal oedema and/or minor fluid collection) were the criteria for diagnosis of acute appendicitis. §Analysed using the chi-squared test. ¶Analysed using one-way ANOVA. #Analysed using the Kruskal–Wallis test. VAS, visual analogue scale; CRP, C-reactive protein.

### Preintervention factors associated with primary non-responsiveness to antibiotics

In the analysis of primary non-responsiveness, there were 832 antibiotic-treated patients available (254 from the APPAC trial and 578 from the APPAC II trial; 349 (42.0%) were women) (*Fig. 1*). Out of the 832 patients, 750 (90.1%) did not undergo surgery within 30 days of randomization and 42 (5.0%) underwent appendectomy for uncomplicated acute appendicitis within 30 days of randomization; 40 (4.8%) were primary non-responders to antibiotics, who underwent appendectomy for complicated acute appendicitis within 30 days of randomization.

In the univariable analysis, an appendiceal diameter ≥15 mm on CT, CRP, leucocyte count, and a body temperature >38°C on admission were associated with primary non-responsiveness to antibiotic therapy. The adjusted RR for primary antibiotic non-responsiveness was 3.52 (95% c.i. 1.84 to 6.74) (*P <* 0.001) for an appendiceal diameter ≥15 mm on admission, 1.07 (95% c.i. 1.02 to 1.13) (*P* = 0.011) for a 10-unit increase in CRP on admission, 1.10 (95% c.i. 1.03 to 1.18) (*P* = 0.005) for leucocyte count on admission, and 2.73 (95% c.i. 1.28 to 5.82) (*P* = 0.010) for a body temperature >38°C on admission. The RRs for univariable analysis for other evaluated factors were not statistically significant and all RRs are presented in detail in *Table 2*. The multivariable model results were very similar to the univariable analysis (*Table 3*).

**Table 2:** Univariable analysis assessing factors predicting primary non-responsiveness to antibiotics in uncomplicated acute appendicitis

Factor*	RR (95% c.i.)	*P*
Appendiceal diameter on CT imaging ≥15 mm	3.52 (1.84,6.74)	< 0.001
CRP†	1.07 (1.02,1.13)	0.011
Leucocyte count	1.10 (1.03,1.18)	0.005
Body temperature >38°C	2.73 (1.28,5.82)	0.010
Age	1.02 (0.99,1.05)	0.120
Sex	0.98 (0.54,1.80)	0.960
VAS score for pain on admission	1.13 (0.98,1.31)	0.086
**Symptom duration (h)**		0.646
Over 18 *versus* 0–6	3.30 (0.45,24.13)	
Over 18 *versus* >6–<12	0.72 (0.35,2.05)	
Over 18 *versus* >12–18	0.95 (0.45,2.00)	

^*^Factors associated with antibiotic non-responsiveness were analysed using a log-binomial model. †Ratio defined for a 10-unit increase. RR, risk ratio; CRP, C-reactive protein; VAS, visual analogue scale.

**Table 3:** Multivariable analysis assessing factors predicting primary non-responsiveness to antibiotics in uncomplicated acute appendicitis

Factor*	RR (95% c.i.)	*P*
Appendiceal diameter on CT imaging ≥15 mm	4.00 (2.00,7.92)	<0.001
CRP†	1.08 (1.03,1.15)	0.005
Leucocyte count	1.10 (1.02,1.19)	0.017
Body temperature >38°C	2.76 (1.27,6.03)	0.011

^*^Factors associated with antibiotic non-responsiveness were analysed using a log-binomial model. †Ratio defined for a 10-unit increase. RR, risk ratio; CRP, C-reactive protein.

### Immediate (6–30 h) postinterventional factors associated with primary non-responsiveness to antibiotics

**Fig. 2:**
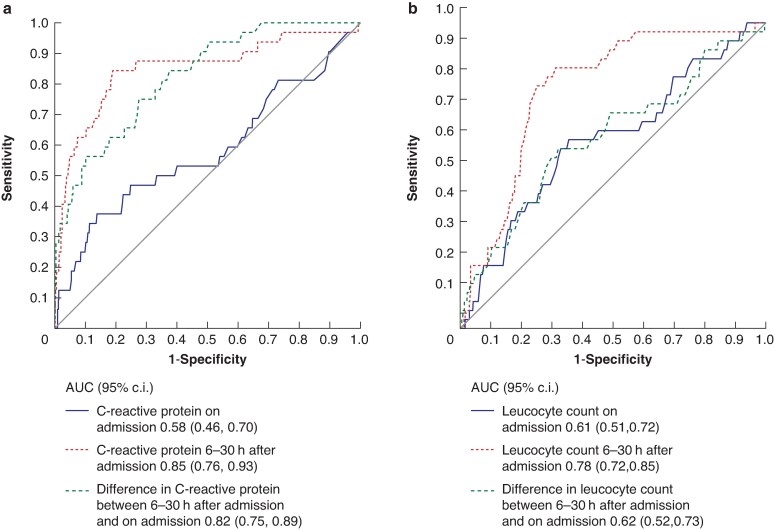
ROC curves for CRP and leucocyte count on admission and during the first 6–30 h after admission, and the change in CRP and leucocyte count during the first day ROC, receiver operating characteristic; CRP, C-reactive protein.

The patient population analysed during the first 6–30 h after admission was similar to the patient population at the time of admission. The ROC curves for CRP and leucocyte count on admission and during the first 6–30 h after admission, and the change in CRP and leucocyte count during the first day are presented in *Fig. 2*. CRP and leucocyte count measured 6–30 h after admission had best clinical value (AUC 0.85 (95% c.i. 0.76 to 0.93) and 0.78 (95% c.i. 0.72 to 0.85) respectively). The sensitivity, specificity, and negative predictive value were 75.0% (95% c.i. 60.0% to 90.0%), 84.2% (95% c.i. 81.5% to 86.8%), and 98.7% (95% c.i. 97.9% to 99.6%) respectively for CRP ≥100 mg/l and 82.4% (95% c.i. 69.5% to 95.2%), 69.8% (95% c.i. 66.5% to 73.2%), and 98.8% (95% c.i. 97.9% to 99.8%) respectively for leucocyte count ≥9 × 10^9^/l. CRP ≥100 mg/l and leucocyte count ≥9 × 10^9^/l during the first 6–30 h after admission were associated with primary non-responsiveness to antibiotic therapy; in univariable analysis the adjusted RR was 14.52 (95% c.i. 6.72 to 31.41) (*P* < 0.001) and 10.09 (95% c.i. 4.24 to 24.02) (*P* < 0.001) respectively and in the multivariable model the adjusted RR was 8.29 (95% c.i. 3.69 to 18.63) (*P* < 0.001) and 4.44 (95% c.i. 1.79 to 11.05) (*P* = 0.001) respectively.

### Factors predicting recurrent appendicitis

In the analysis of recurrent appendicitis, 732 patients (mean(s.d.) age of 36(12) years) were available, 229 (mean(s.d.) age of 36(12) years) from the APPAC trial^19^ and 503 (mean(s.d.) age of 35(12) years) from the APPAC II trial^21^; 298 (40.7%) were women (*Fig. 1*). Out of all 732 patients, 192 (26.2%) underwent appendectomy for true recurrent appendicitis during the 3-year follow-up; 167 (87.0%) had uncomplicated acute appendicitis at surgery and 25 (13.0%) had complicated acute appendicitis at surgery. The appendectomy rate at 3 years was 27.8% (208 of 748). There were no statistically significant findings in the factors evaluated for predicting appendicitis recurrence and the results are presented in detail in *Table 4*.

**Table 4:** Analysis assessing prognostic factors for appendicitis recurrence after initial antibiotic treatment

Factor*	HR (95% c.i.)†	*P*
Age	1.00 (0.99,1.01)	0.934
Sex	0.93 (0.70,1.25)	0.642
VAS score for pain on admission	1.03 (0.97,1.10)	0.327
Leucocyte count	1.02 (0.98,1.05)	0.370
Body temperature	1.18 (0.62,2.24)	0.611
CRP	1.00 (0.99,1.00)	0.211
Appendiceal diameter on CT imaging	1.05 (0.99,1.11)	0.101

^*^The prognostic factors for recurrent appendicitis were evaluated using a Cox regression model. †Trial group (APPAC, APPAC II—intravenous antibiotics followed by oral antibiotics, and APPAC II—only oral antibiotics) included in the analysis. VAS, visual analogue scale; CRP, C-reactive protein.

## Discussion

In this analysis assessing primary non-responsiveness to initial antibiotics in CT-diagnosed uncomplicated acute appendicitis, patients with an appendiceal diameter ≥15 mm or a body temperature >38°C on admission were found to have an increased risk of primary antibiotic non-responsiveness. In addition, immediate postintervention findings of CRP ≥100 mg/l and leucocyte count ≥9 × 10^9^/l during the first 6–30 h after admission were also found to be associated with primary antibiotic non-responsiveness. CRP at a cut-off of ≥100 mg/l at 6–30 h after initiation of antibiotics yielded a negative predictive value of 99%; thus, for CRP <100 mg/l there is a 99% probability of successful primary antibiotic treatment. There were no prognostic factors for appendicitis recurrence.

Oral antibiotics for uncomplicated acute appendicitis enable possible outpatient management, resulting in substantial cost savings and benefits in the use of hospital resources. In the large CODA trial, outpatient management of appendicitis was already proven safe, despite the inclusion of patients with a more complicated course of acute appendicitis, as approximately half of the participants (46.1%) randomized to receive antibiotics in the CODA trial underwent outpatient management with hospital discharge within 24 h without an increased rate of complications compared with those patients admitted to the hospital^12^. Safe outpatient management highlights the need for accurate patient selection and the importance of choosing the optimal primary treatment. Identifying patients at higher risk of appendectomy after antibiotic treatment could be valuable for guiding treatment decisions. The finding of CRP ≥100 mg/l during the first 6–30 h after admission having a negative predictive value of 99% can be considered an accurate objective measure when evaluating the effectiveness of treatment shortly after initiation of antibiotics. Considering the variability of patient symptom resolution, such an objective measure would be helpful to aid clinicians in the assessment of the effectiveness of an initiated treatment when there is ambiguity, with the potential to decrease the number of unnecessary appendectomies due to uncertainty related to the initiated non-operative approach.

The on-admission findings of an appendiceal diameter ≥15 mm and a body temperature >38°C were prognostic factors for non-responsiveness in this study, corroborating previously reported studies^13,16^. Patients with a larger appendiceal diameter may not be optimal candidates for non-operative management and these patients should be considered for emergency appendectomy. The large CODA trial found that female sex was associated with an increased risk of appendectomy within the first 30 days of initial antibiotics^13^, in contrast to this study, where female sex did not increase the risk of appendectomy for complicated acute appendicitis, that is true non-responsiveness, highlighting the need for more disease-related objective tools to assess the effectiveness of initiated treatments. Besides single characteristics evaluating appendicitis severity, efforts have been made towards a composite endpoint. The recently published Scoring System of Appendicitis Severity (SAS) 2.0^22^ assesses the probability of having complicated disease by combining objective clinical, laboratory, and imaging findings, and one subjective parameter of numeric pain rating scale. However, SAS 2.0 does not provide any treatment recommendations; it is merely a tool for improving the differential diagnosis of appendicitis severity. Wider appendiceal diameter, higher body temperature, and the presence of an appendicolith are taken into account in SAS 2.0^22^.

Given the substantial evidence from multiple RCTs supporting the safety and efficacy of antibiotics for acute appendicitis^7,15,23,24^, the present study also aimed to address the knowledge gap regarding potential prognostic factors for appendicitis recurrence. The exact mechanisms leading to recurrent appendicitis are unknown, but it seems likely that recurrence is a multifactorial issue potentially including microbiological and immunological factors. Among patients with uncomplicated and complicated acute appendicitis differences in appendiceal microbiome have already been described^25^, but their potential role in appendicitis recurrence is unclear. In this study of patients treated with antibiotics for uncomplicated acute appendicitis, no clinical findings were predictive of appendicitis recurrence. In the recent cohort study from the CODA trial^26^ assessing factors associated with recurrent appendicitis, even the factors commonly related to appendicitis severity were not strongly associated with an increased risk of appendectomy in long-term follow-up after successful initial antibiotic treatment. Larger collaboration with extensive international scientific research on uncomplicated acute appendicitis has already been initiated and an individual patient data meta-analysis comparing antibiotics with appendicectomy for the treatment of uncomplicated acute appendicitis has been published^10^. This has contributed to establishing uniform and standardized criteria for distinguishing between uncomplicated and complicated instances of acute appendicitis before choosing the optimal treatment, but international collaboration could also assist in identifying preinterventional factors that predict appendicitis recurrence.

One concern with increasing non-operative management for uncomplicated acute appendicitis is the remaining uncertainty of possible undetected appendiceal neoplasms. However, the risk of appendiceal neoplasms is directly associated with appendicitis severity, underlining the importance of differentiating between uncomplicated and complicated acute appendicitis. The reported appendiceal tumour risk associated with complicated acute appendicitis (2.4%^3^) has been reported to be markedly higher compared with uncomplicated acute appendicitis (1.5%)^3,27–30^ with the highest prevalence of up to 14.3% in complicated acute appendicitis presenting with a periappendicular abscess in a recent large prospective cohort study^3^. These findings mitigate the risk of missed appendiceal neoplasms associated with antibiotic treatment of uncomplicated acute appendicitis.

This study has several limitations. The first limitation of this study is the relatively small number of patients with non-responsiveness to the antibiotics. This is due to the careful initial inclusion and exclusion criteria for the RCTs assessing specifically patients with CT-diagnosed uncomplicated acute appendicitis and demonstrates a strength of the initial RCT patient selection according to the study hypothesis. Second, the antibiotic treatment between the three groups varied. However, considering the similar characteristics of all three groups and the two trials, the identical inclusion criteria in both the APPAC trial and the APPAC II trial, and consistent results at 1-, 2-, and 3-year follow-up, the variability of antibiotic treatments most likely has no clinical importance. Furthermore, recently even the need for antibiotics in uncomplicated acute appendicitis has been questioned^17,31,32^. In fact, if future research shows that uncomplicated acute appendicitis may resolve without antibiotics, the results of this study might offer valuable tools to aid in the selection of patients suitable for symptomatic treatment. The strengths of this trial include a large, randomized patient population with both a high follow-up rate and as accurate as possible diagnosis of appendicitis severity after appendectomy. In addition, the assessment for primary non-responders is as accurate as possible, as all of the patients operated on for uncomplicated acute appendicitis were excluded from the non-responder group.

In conclusion, this secondary analysis of two RCTs assessing a large population of patients initially treated with antibiotics found an appendiceal diameter ≥15 mm and a body temperature >38°C on admission to be associated with non-responsiveness to antibiotics for uncomplicated acute appendicitis. After initiation of antibiotics, CRP ≥100 mg/l and leucocyte count ≥9 × 10^9^/l during the first 6–30 h after admission showed an association with non-responsiveness to antibiotics. Conversely, patients with CRP <100 mg/l at 24 h of antibiotic treatment for uncomplicated acute appendicitis have a 99% likelihood of successful non-operative primary management. These findings can aid clinicians and patients to optimize the primary joint treatment decisions and assess the effectiveness of the initiated non-operative treatment in CT-confirmed uncomplicated acute appendicitis.

## Supplementary Material

znaf143_Supplementary_Data

## Data Availability

Data available from corresponding author for reasonable requests and collaboration.

## References

[1] Bhangu A, Soreide K, Di Saverio S, Assarsson JH, Drake FT. Acute appendicitis: modern understanding of pathogenesis, diagnosis, and management. Lancet 2015;386:1278–1287 26460662 10.1016/S0140-6736(15)00275-5

[2] Livingston EH, Fomby TB, Woodward WA, Haley RW. Epidemiological similarities between appendicitis and diverticulitis suggesting a common underlying pathogenesis. Arch Surg 2011;146:308–314 21422362 10.1001/archsurg.2011.2

[3] Salminen R, Alajaaski J, Rautio T, Hurme S, Nordstrom P, Makarainen E et al Appendiceal tumor prevalence in patients with periappendicular abscess. JAMA Surg 2025;160:526–534 40172884 10.1001/jamasurg.2025.0312 PMC11966475

[4] Fitz RH . Perforating Inflammation of the Vermiform Appendix: With Special Reference to its Early Diagnosis and Treatment. Philadelphia: Wm. J. Dornan, 1886; 31 pages

[5] McBurney C . Experience with early operative interference in cases of disease of the vermiform appendix. N.Y. Med. J 1889;50:676–684

[6] O'Leary DP, Walsh SM, Bolger J, Baban C, Humphreys H, O'Grady S et al A randomized clinical trial evaluating the efficacy and quality of life of antibiotic-only treatment of acute uncomplicated appendicitis: results of the COMMA trial. Ann Surg 2021;274:240–247 33534226 10.1097/SLA.0000000000004785

[7] Salminen P, Tuominen R, Paajanen H, Rautio T, Nordstrom P, Aarnio M et al Five-year follow-up of antibiotic therapy for uncomplicated acute appendicitis in the APPAC randomized clinical trial. JAMA 2018;320:1259–1265 30264120 10.1001/jama.2018.13201 PMC6233612

[8] Talan DA, Saltzman DJ, Mower WR, Krishnadasan A, Jude CM, Amii R et al Antibiotics-first versus surgery for appendicitis: a US pilot randomized controlled trial allowing outpatient antibiotic management. Ann Emerg Med 2017;70:1–11.e19 27974169 10.1016/j.annemergmed.2016.08.446 PMC5616169

[9] Selanne L, Haijanen J, Sippola S, Hurme S, Rautio T, Nordstrom P et al Three-year outcomes of oral antibiotics vs intravenous and oral antibiotics for uncomplicated acute appendicitis: a secondary analysis of the APPAC II randomized clinical trial. JAMA Surg 2024;159:727–735 38630471 10.1001/jamasurg.2023.5947 PMC11024776

[10] Scheijmans JCG, Haijanen J, Flum DR, Bom WJ, Davidson GH, Vons C et al Antibiotic treatment versus appendicectomy for acute appendicitis in adults: an individual patient data meta-analysis. Lancet Gastroenterol Hepatol 2025;10:222–233 39827891 10.1016/S2468-1253(24)00349-2

[11] Ceresoli M, Fumagalli C, Fugazzola P, Zanini N, Magnone S, Ravasi M et al Outpatient non-operative management of uncomplicated acute appendicitis: a non-inferiority study. World J Surg 2023;47:2378–2385 37210423 10.1007/s00268-023-07065-7 PMC10474178

[12] Writing Group for the CODA Collaborative . Analysis of outcomes associated with outpatient management of nonoperatively treated patients with appendicitis. JAMA Netw Open 2022;5:e2220039 35796152 10.1001/jamanetworkopen.2022.20039 PMC9250049

[13] Writing Group for the CODA Collaborative . Patient factors associated with appendectomy within 30 days of initiating antibiotic treatment for appendicitis. JAMA Surg 2022;157:e216900 35019975 10.1001/jamasurg.2021.6900 PMC8756360

[14] Vons C, Barry C, Maitre S, Pautrat K, Leconte M, Costaglioli B et al Amoxicillin plus clavulanic acid versus appendicectomy for treatment of acute uncomplicated appendicitis: an open-label, non-inferiority, randomised controlled trial. Lancet 2011;377:1573–1579 21550483 10.1016/S0140-6736(11)60410-8

[15] Collaborative C, Davidson GH, Flum DR, Monsell SE, Kao LS, Voldal EC et al Antibiotics versus appendectomy for acute appendicitis—longer-term outcomes. N Engl J Med 2021;385:2395–2397 34694761 10.1056/NEJMc2116018

[16] Haijanen J, Sippola S, Loyttyniemi E, Hurme S, Gronroos J, Rautio T et al Factors associated with primary nonresponsiveness to antibiotics in adults with uncomplicated acute appendicitis: a prespecified secondary analysis of a randomized clinical trial. JAMA Surg 2021;156:1179–1181 34613361 10.1001/jamasurg.2021.5003 PMC8495607

[17] Lund H, Haijanen J, Suominen S, Hurme S, Sippola S, Rantanen T et al A randomized double-blind noninferiority clinical multicenter trial on oral moxifloxacin versus placebo in the outpatient treatment of uncomplicated acute appendicitis: APPAC IV study protocol. Scand J Surg 2024;114:3–12 39636024 10.1177/14574969241293018

[18] Sippola S, Haijanen J, Viinikainen L, Gronroos J, Paajanen H, Rautio T et al Quality of life and patient satisfaction at 7-year follow-up of antibiotic therapy vs appendectomy for uncomplicated acute appendicitis: a secondary analysis of a randomized clinical trial. JAMA Surg 2020;155:283–289 32074268 10.1001/jamasurg.2019.6028 PMC7042917

[19] Salminen P, Paajanen H, Rautio T, Nordstrom P, Aarnio M, Rantanen T et al Antibiotic therapy vs appendectomy for treatment of uncomplicated acute appendicitis: the APPAC randomized clinical trial. JAMA 2015;313:2340–2348 26080338 10.1001/jama.2015.6154

[20] Sippola S, Haijanen J, Gronroos J, Rautio T, Nordstrom P, Rantanen T et al Effect of oral moxifloxacin vs intravenous ertapenem plus oral levofloxacin for treatment of uncomplicated acute appendicitis: the APPAC II randomized clinical trial. JAMA 2021;325:353–362 33427870 10.1001/jama.2020.23525 PMC7802006

[21] Haijanen J, Sippola S, Gronroos J, Rautio T, Nordstrom P, Rantanen T et al Optimising the antibiotic treatment of uncomplicated acute appendicitis: a protocol for a multicentre randomised clinical trial (APPAC II trial). BMC Surg 2018;18:117 30558607 10.1186/s12893-018-0451-y PMC6296129

[22] Scheijmans JCG, Bom WJ, Ghori UH, van Geloven AAW, Hannink G, van Rossem CC et al Development and validation of the scoring system of appendicitis severity 2.0. JAMA Surg 2024;159:642–649 38536188 10.1001/jamasurg.2024.0235 PMC10974687

[23] Collaborative C, Flum DR, Davidson GH, Monsell SE, Shapiro NI, Odom SR et al A randomized trial comparing antibiotics with appendectomy for appendicitis. N Engl J Med 2020;383:1907–1919 33017106 10.1056/NEJMoa2014320

[24] Moris D, Paulson EK, Pappas TN. Diagnosis and management of acute appendicitis in adults: a review. JAMA 2021;326:2299–2311 34905026 10.1001/jama.2021.20502

[25] Vanhatalo S, Munukka E, Kallonen T, Sippola S, Gronroos J, Haijanen J et al Appendiceal microbiome in uncomplicated and complicated acute appendicitis: a prospective cohort study. PLoS One 2022;17:e0276007 36240181 10.1371/journal.pone.0276007 PMC9565418

[26] Writing Group for the CODA Collaborative . Factors associated with recurrent appendicitis after successful treatment with antibiotics. Br J Surg 2023;110:1482–1489 37459231 10.1093/bjs/znad218 PMC10564398

[27] Alajaaski J, Lietzen E, Gronroos JM, Mecklin JP, Leppaniemi A, Nordstrom P et al The association between appendicitis severity and patient age with appendiceal neoplasm histology-a population-based study. Int J Colorectal Dis 2022;37:1173–1180 35474547 10.1007/s00384-022-04132-8 PMC9072484

[28] Brunner M, Lapins P, Langheinrich M, Baecker J, Krautz C, Kersting S et al Risk factors for appendiceal neoplasm and malignancy among patients with acute appendicitis. Int J Colorectal Dis 2020;35:157–163 31811385 10.1007/s00384-019-03453-5

[29] Lietzen E, Gronroos JM, Mecklin JP, Leppaniemi A, Nordstrom P, Rautio T et al Appendiceal neoplasm risk associated with complicated acute appendicitis-a population based study. Int J Colorectal Dis 2019;34:39–46 30242478 10.1007/s00384-018-3156-x

[30] Peltrini R, Cantoni V, Green R, Lionetti R, D'Ambra M, Bartolini C et al Risk of appendiceal neoplasm after interval appendectomy for complicated appendicitis: a systematic review and meta-analysis. Surgeon 2021;19:e549–e558 33640282 10.1016/j.surge.2021.01.010

[31] Park HC, Kim MJ, Lee BH. Randomized clinical trial of antibiotic therapy for uncomplicated appendicitis. Br J Surg 2017;104:1785–1790 28925502 10.1002/bjs.10660

[32] Salminen P, Sippola S, Haijanen J, Nordstrom P, Rantanen T, Rautio T et al Antibiotics *versus* placebo in adults with CT-confirmed uncomplicated acute appendicitis (APPAC III): randomized double-blind superiority trial. Br J Surg 2022;109:503–509 35576384 10.1093/bjs/znac086 PMC10364767

