# Supplementary material for: Prognostic factors associated with primary non-responsiveness to antibiotics and appendicitis recurrence for CT-diagnosed uncomplicated acute appendicitis: secondary analysis of two randomized clinical trials
Source: Br J Surg. 2025 Jul 31;112(7):znaf143. doi: 10.1093/bjs/znaf143 (PMC12311427; doi:10.1093/bjs/znaf143)
Supplement: znaf143_Supplementary_Data [file znaf143_supplementary_data.docx]

**Supplementary material**

**Prognostic factors associated with primary non-responsiveness to antibiotics and appendicitis recurrence for CT-diagnosed uncomplicated acute appendicitis: A secondary analysis of two randomized clinical trials**

Selänne Liisa, MD^1,2^, Hurme Saija, MSc^3^, Sippola Suvi, MD, PhD^4^, Rautio Tero, MD, PhD^5,6^, Nordström Pia, MD, PhD^7,8^, Rantanen Tuomo, MD, PhD^9,10^, Pinta Tarja, MD, PhD^11^, Ilves Imre, MD, PhD^12^, Mattila Anne, MD, PhD^13^, Sävelä Eeva-Liisa, MD^14^, Rintala Jukka, MD, PhD^5,15^, Paajanen Hannu, MD, PhD^12^, Grönroos Juha, MD, PhD^1,2^, *Haijanen Jussi, MD, PhD^1,2^, *Salminen Paulina, MD, PhD^1,2^

*Equal contribution

Affiliations:

1. Division of Digestive Surgery and Urology, Turku University Hospital, Turku, Finland
2. Department of Surgery, University of Turku, Turku, Finland
3. Department of Biostatistics, University of Turku and Turku University Hospital, Turku, Finland
4. Department of Surgery, Helsinki University Hospital, Helsinki, Finland
5. Department of Surgery, Oulu University Hospital, Oulu, Finland
6. Medical Research Center Oulu, University of Oulu, Finland
7. Department of Gastroenterology and Alimentary tract surgery, Tampere University Hospital
8. Faculty of Medicine and Health Technology, University of Tampere, Tampere, Finland
9. Department of Surgery, Kuopio University Hospital, Kuopio, Finland
10. Department of Surgery, Institute of Clinical Medicine, University of Eastern Finland.
11. Department of Surgery, Seinäjoki Central Hospital, Seinäjoki, Finland
12. Department of Surgery, Mikkeli Central Hospital, Mikkeli, Finland
13. Wellbeing Services County of Central Finland / Hospital Nova of Central Finland
14. Department of Surgery, Satakunta Central Hospital, Pori, Finland
15. Department of Surgery, Rovaniemi Central Hospital, Rovaniemi, Finland

**Corresponding author**:

Paulina Salminen, MD, PhD, Professor of Surgery

[paulina.salminen@tyks.fi](mailto:paulina.salminen@tyks.fi)

University of Turku, Department of Surgery

Turku University Hospital, Division of Digestive Surgery and Urology

P.O. Box 52

20520 Turku, Finland

Supplement 1. The APPAC trial protocol

Supplement 2. The APPAC Statistical analysis plan

Supplement 3. The APPACII trial protocol

Supplement 4. The APPACII Statistical analysis plan

**Supplement 1. The APPAC trial protocol**

**Supplement 2. APPAC trial: Statistical analysis plan**

**APPAC trial protocol and statistical analysis plan**

**Original trial protocol + SAP:** 21.4.2009 Ethical committee ETMK: 37/180/2009 144§

(EudraCT: 2009-017626-39)

Amendments to trial protocol:

1. QOL assessment 23.1.2018 ETMK: 37/180/2009 18§
2. MRI imaging for patients with an intact appendix 27.8.2019 ETMK: 37/180/2009 381

**APPAC trial: statistical analysis plan (original)**

Paulina Salminen, M.D., Ph.D., Hannu Paajanen, M.D., Ph.D., Tero Rautio, M.D., Ph.D., Saija Hurme, M.Sc., and Juha M. Grönroos, M.D., Ph.D.,

for the Finnish APPAC group

**1. Introduction**

The APPAC trial is a randomized prospective controlled, open label, non-inferiority multicenter trial designed to compare antibiotic therapy (ertapenem) with emergency appendectomy in the treatment of uncomplicated AA. The primary endpoint of the study is the success of the randomized treatment. In the antibiotic treatment arm successful treatment is defined as being discharged from the hospital without the need for surgical intervention and no recurrent appendicitis during a follow-up of one-year (treatment efficacy). Treatment efficacy in the operative treatment arm is defined as successful appendectomy evaluated to be 100 %. Secondary endpoints are post-intervention complications, overall morbidity and mortality, the length of hospital stay and sick leave, treatment costs and pain scores (VAS, visual analoque scale).

**2. Sample size calculation**

The sample size calculation of the trial was based on the self-evident fact that the efficacy of appendectomy as a treatment for AA is 100 %, but antibiotic therapy will not provide adequate source control in all patients with uncomplicated AA. However, the hypothesis of the APPAC trial is that operative treatment of uncomplicated AA is not mandatory for the majority of patients as 75 – 85 % of patients with uncomplicated AA can be cured with wide-spectrum antibiotics avoiding a large number of unnecessary appendectomies ^1^. For the primary endpoint of treatment success for the randomized therapy tested in a randomized, controlled, open label, non-inferiority multicenter trial, we assumed 99 % healing rate of AA in the appendectomy group vs. 80 % success rate for the antibiotic therapy. A non-inferiority margin of 24 percentage points was used in the sample size calculations meaning that the lower limit of the success in antibiotic therapy would be 75 %. We calculated that a sample size of 275 patients per group would give a power of 0.9 (1-β) to establish whether antibiotic treatment was not inferior to appendectomy evaluated by treatment success in both study arms (significance level of 0.05 α). With an estimated 10 percent of the trial patients lost to follow-up, a maximum of 610 patients will be enrolled.

**3. Interim analyses**

To ensure the safety of the antibiotic treatment the frequency of the treatment efficacy will be examined after randomizing 150-200 patients. No statistical tests will be conducted at this point.

**4. Statistical analysis**

Categorical variables will be characterized by treatment using frequencies and percents and for continuous variables means and standard deviations or medians and range will be used. Two-tailed p-values will be used and p-values less than 0.05 will be considered statistically significant. The main analyses will be based on the intention-to-treat principle. Missing data will be excluded from the analyses. Statistical analyses will be performed using SAS System for Windows, Version 9.2 (SAS Institute Inc., Cary, NC).

**4.1. Primary outcome**

Primary endpoint of the study is treatment efficacy and groups will be compared using equivalence trial setting. For comparing treatments the 95% confidence intervals for treatment efficacy will be presented.

**4.2. Secondary outcomes**

For the secondary endpoints data will be compared as superiority trial setting and in superiority tests a two-tailed P value ≤ 0.05 will be considered statistically significant. For categorical variables Pearson’s Chi Squared test will be used to test difference between groups. Differences between groups for normally distributed variables will be tested using independent sample t-test and for non-normally distributed variables Mann-Whitney U- test will be used. Normality tests will be performed to justify the analyses.

**4.3. Subgroup analyses**

Additional analyses will be performed for overall morbidity using per-protocol principle.

**5. Cost analysis**

All related costs will be estimated based on the actual input terms of resource use and personnel in the 12-month follow-up period after randomization. All costs will be derived from the Finnish hospital cost or determined in co-operation with the hospital administration. Direct medical costs will be recorded in the case record forms. Indirect costs arising from losses in productivity will be assessed by means of the Health and Labor questionnaire and will be calculated by means of the friction cost method.

**6. Follow-up**

The main results of the trial will be reported after one year follow-up of the patients. After that the patients will be interviewed also at 3, 5 and 10 years after treatment and the data is evaluated in every time-point.

**References**

Mason, R.J., A. Moazzez, H. Sohn, and N. Katkhouda, Meta-analysis of randomized trials comparing antibiotic therapy with appendectomy for acute uncomplicated (no abscess or phlegmon) appendicitis*.* Surg Infect (Larchmt), 2012. 13(2): p. 74-84.

**APPAC trial: statistical analysis plan (5-year follow-up)**

Paulina Salminen, M.D., Ph.D., Hannu Paajanen, M.D., Ph.D., Tero Rautio, M.D., Ph.D., Pia Nordström, M.D., Ph.D., Markku Aarnio, M.D., Ph.D., Tuomo Rantanen, M.D., Ph.D., Risto Tuominen, M.P.H., Ph.D, Saija Hurme, M.Sc., Johanna Virtanen, M.D., Jukka-Pekka Mecklin, M.D., Ph.D., Juhani Sand, M.D., Ph.D., Airi Jartti, M.D., Irina Rinta-Kiikka, M.D., Ph.D., and Juha M. Grönroos, M.D., Ph.D.,

for the Finnish APPAC group

From the Division of Digestive Surgery and Urology, Departments of Acute and Digestive Surgery, Turku University Hospital and The Department of Surgery, Turku University, Turku (P.S., J.M.G.), the Department of Surgery, Mikkeli Central Hospital, Mikkeli (H.P.) and Institute of Clinical Medicine, University of Eastern Finland (H.P., J-P.M., T. Rantanen), the Department of Surgery, Oulu University Hospital, Oulu (T. Rautio), Division of Surgery, Gastroenterology and Oncology, Tampere University Hospital, Tampere (P.N., J.S.), Department of Surgery, Jyväskylä Central Hospital, Jyväskylä (M.A., J-P.M.), Department of Surgery, Kuopio University Hospital, Kuopio and the Department of Surgery, Seinäjoki Central Hospital, Seinäjoki (T. Rantanen), Department of Public Health, University of Turku, and Primary Health Care Unit, Hospital District of Southwest Finland (R.T.), Department of Biostatistics, University of Turku, Turku (S.H.), Department of Radiology, Turku University Hospital, Turku (J.V.), Department of Radiology, Oulu University Hospital, Oulu (A.J.), Department of Radiology, Tampere University Hospital, Tampere (I.R-K.) – all in Finland.

**1. Introduction**

The APPAC trial is a randomized prospective controlled, open label, non-inferiority multicenter trial designed to compare antibiotic therapy (ertapenem) with emergency appendectomy in the treatment of uncomplicated AA. The primary endpoint of the study is the success of the randomized treatment. In the antibiotic treatment arm successful treatment is defined as being discharged from the hospital without the need for surgical intervention and no recurrent appendicitis during a follow-up of one-year (treatment efficacy). Treatment efficacy in the operative treatment arm is defined as successful appendectomy evaluated to be 100 %. Secondary endpoints are post-intervention complications, overall morbidity and mortality, the length of hospital stay and sick leave, treatment costs and pain scores (VAS, visual analoque scale).

**2. Sample size calculation**

The sample size calculation of the trial was based on the self-evident fact that the efficacy of appendectomy as a treatment for AA is 100 %, but antibiotic therapy will not provide adequate source control in all patients with uncomplicated AA. However, the hypothesis of the APPAC trial is that operative treatment of uncomplicated AA is not mandatory for the majority of patients as 75 – 85 % of patients with uncomplicated AA can be cured with wide-spectrum antibiotics avoiding a large number of unnecessary appendectomies ^1^. For the primary endpoint of treatment success for the randomized therapy tested in a randomized, controlled, open label, non-inferiority multicenter trial, we assumed 99 % healing rate of AA in the appendectomy group vs. 80 % success rate for the antibiotic therapy. A non-inferiority margin of 24 percentage points was used in the sample size calculations meaning that the lower limit of the success in antibiotic therapy would be 75 %. We calculated that a sample size of 275 patients per group would give a power of 0.9 (1-β) to establish whether antibiotic treatment was not inferior to appendectomy evaluated by treatment success in both study arms (significance level of 0.05 α). With an estimated 10 percent of the trial patients lost to follow-up, a maximum of 610 patients will be enrolled.

**3. Interim analyses**

To ensure the safety of the antibiotic treatment the frequency of the treatment efficacy will be examined after randomizing 150-200 patients. No statistical tests will be conducted at this point.

**4. Statistical analysis**

Categorical variables will be characterized by treatment using frequencies and percents and for continuous variables means and standard deviations or medians and range will be used. Two-tailed p-values will be used and p-values less than 0.05 will be considered statistically significant. The main analyses will be based on the intention-to-treat principle. Missing data will be excluded from the analyses. Statistical analyses will be performed using SAS System for Windows, Version 9.2 (SAS Institute Inc., Cary, NC).

**4.1. Primary outcome**

Primary endpoint of the study is treatment efficacy and groups will be compared using equivalence trial setting. For comparing treatments the 95% confidence intervals for treatment efficacy will be presented.

**4.2. Secondary outcomes**

For the secondary endpoints data will be compared as superiority trial setting and in superiority tests a two-tailed P value ≤ 0.05 will be considered statistically significant. For categorical variables Pearson’s Chi Squared test will be used to test difference between groups. Differences between groups for normally distributed variables will be tested using independent sample t-test and for non-normally distributed variables Mann-Whitney U- test will be used. Normality tests will be performed to justify the analyses.

**4.3. Subgroup analyses**

Additional analyses will be performed for overall morbidity using per-protocol principle.

In order to find predictive factors for a more complicated course of appendicitis a subgroup of patients with a complicated AA will be compared with the rest of the patients in antibiotic group. First the explorative analyses will be performed for pre-intervention variables (CRP, Hb, leukocyte, creatinine and pain) and if statistically significant differences between groups will be found, then ROC-analysis will be performed to find a cut-point value for variable in order to predict complicated AA. A ROC-curve, sensitivity and specificity will be presented as the results of analyses.

**5. Cost analysis**

All related costs will be estimated based on the actual input terms of resource use and personnel in the 12-month follow-up period after randomization. All costs will be derived from the Finnish hospital cost or determined in co-operation with the hospital administration. Direct medical costs will be recorded in the case record forms. Indirect costs arising from losses in productivity will be assessed by means of the Health and Labor questionnaire and will be calculated by means of the friction cost method.

**6. Follow-up**

The main results of the trial will be reported after one year follow-up of the patients. After that the patients will be interviewed also at 3, 5 and 10 years after treatment and the data is evaluated in every time-point.

**References**

Mason, R.J., A. Moazzez, H. Sohn, and N. Katkhouda, **Meta-analysis of randomized trials comparing antibiotic therapy with appendectomy for acute uncomplicated (no abscess or phlegmon) appendicitis***.* Surg Infect (Larchmt), 2012. **13**(2): p. 74-84.

**Summary of changes**

**Amendment for chapter 4.3. Subgroup analyses**

In order to find predictive factors for a more complicated course of appendicitis a subgroup of patients with a complicated AA will be compared with the rest of the patients in antibiotic group. First the explorative analyses will be performed for pre-intervention variables (CRP, Hb, leukocyte, creatinine and pain) and if statistically significant differences between groups will be found, then ROC-analysis will be performed to find a cut-point value for variable in order to predict complicated AA. A ROC-curve, sensitivity and specificity will be presented as the results.

**APPAC trial: statistical analysis plan (final)**

Paulina Salminen, M.D., Ph.D., Roosa Salminen, M.D, Hannu Paajanen, M.D., Ph.D., Tero Rautio, M.D., Ph.D., Pia Nordström, M.D., Ph.D., Markku Aarnio, M.D., Ph.D., Tuomo Rantanen, M.D., Ph.D., Risto Tuominen, M.P.H., Ph.D, Saija Hurme, M.Sc., Johanna Virtanen, M.D., Jukka-Pekka Mecklin, M.D., Ph.D., Juhani Sand, M.D., Ph.D., Airi Jartti, M.D., Irina Rinta-Kiikka, M.D., Ph.D., and Juha M. Grönroos, M.D., Ph.D.,

for the Finnish APPAC group

From the Division of Digestive Surgery and Urology, Departments of Acute and Digestive Surgery, Turku University Hospital and The Department of Surgery, Turku University, Turku (P.S., J.M.G.), the Department of Surgery, Mikkeli Central Hospital, Mikkeli (H.P.) and Institute of Clinical Medicine, University of Eastern Finland (H.P., J-P.M., T. Rantanen), Oulu University Hospital and Medical Research Center Oulu, University of Oulu (R.S., T. Rautio), Division of Surgery, Gastroenterology and Oncology, Tampere University Hospital, Tampere (P.N., J.S.), Department of Surgery, Hospital Nova of Central Finland, Jyväskylä (M.A., J-P.M.), Department of Surgery, Kuopio University Hospital, Kuopio and the Department of Surgery, Seinäjoki Central Hospital, Seinäjoki (T. Rantanen), Department of Public Health, University of Turku, and Primary Health Care Unit, Hospital District of Southwest Finland (R.T.), Department of Biostatistics, University of Turku and Turku University Hospital, Turku (S.H.), Department of Radiology, Turku University Hospital, Turku (J.V.), Department of Radiology, Oulu University Hospital, Oulu (A.J.), Department of Radiology, Tampere University Hospital, Tampere (I.R-K.) – all in Finland.

**1. Introduction**

The APPAC trial is a randomized prospective controlled, open label, non-inferiority multicenter trial designed to compare antibiotic therapy (ertapenem) with emergency appendectomy in the treatment of uncomplicated AA. The primary endpoint of the study is the success of the randomized treatment. In the antibiotic treatment arm successful treatment is defined as being discharged from the hospital without the need for surgical intervention and no recurrent appendicitis during a follow-up of one-year (treatment efficacy). Treatment efficacy in the operative treatment arm is defined as successful appendectomy evaluated to be 100 %. Secondary endpoints are post-intervention complications, overall morbidity and mortality, the length of hospital stay and sick leave, treatment costs and pain scores (VAS, visual analoque scale).

**2. Sample size calculation**

The sample size calculation of the trial was based on the self-evident fact that the efficacy of appendectomy as a treatment for AA is 100 %, but antibiotic therapy will not provide adequate source control in all patients with uncomplicated AA. However, the hypothesis of the APPAC trial is that operative treatment of uncomplicated AA is not mandatory for the majority of patients as 75 – 85 % of patients with uncomplicated AA can be cured with wide-spectrum antibiotics avoiding a large number of unnecessary appendectomies^3^. For the primary endpoint of treatment success for the randomized therapy tested in a randomized, controlled, open label, non-inferiority multicenter trial, we assumed 99 % healing rate of AA in the appendectomy group vs. 80 % success rate for the antibiotic therapy. A non-inferiority margin of 24 percentage points was used in the sample size calculations meaning that the lower limit of the success in antibiotic therapy would be 75 %. We calculated that a sample size of 275 patients per group would give a power of 0.9 (1-β) to establish whether antibiotic treatment was not inferior to appendectomy evaluated by treatment success in both study arms (significance level of 0.05 α). With an estimated 10 percent of the trial patients lost to follow-up, a maximum of 610 patients will be enrolled.

**3. Interim analyses**

To ensure the safety of the antibiotic treatment the frequency of the treatment efficacy will be examined after randomizing 150-200 patients. No statistical tests will be conducted at this point.

**4. Statistical analysis**

Categorical variables will be characterized by treatment using frequencies and percents and for continuous variables means and standard deviations or medians and range will be used. Two-tailed p-values will be used and p-values less than 0.05 will be considered statistically significant. The main analyses will be based on the intention-to-treat principle. Missing data will be excluded from the analyses. Statistical analyses will be performed using SAS System for Windows, Version 9.2 (SAS Institute Inc., Cary, NC).

**4.1. Primary outcome**

Primary endpoint of the study is treatment efficacy and groups will be compared using equivalence trial setting. For comparing treatments the 95% confidence intervals for treatment efficacy will be presented.

**4.2. Secondary outcomes**

For the secondary endpoints data will be compared as superiority trial setting and in superiority tests a two-tailed P value ≤ 0.05 will be considered statistically significant. For categorical variables Pearson’s Chi Squared test will be used to test difference between groups. Differences between groups for normally distributed variables will be tested using independent sample t-test and for non-normally distributed variables Mann-Whitney U- test will be used. Normality tests will be performed to justify the analyses.

**4.3. Subgroup analyses**

Additional analyses will be performed for overall morbidity using per-protocol principle.

In order to find predictive factors for a more complicated course of appendicitis a subgroup of patients with a complicated AA will be compared with the rest of the patients in antibiotic group. First the explorative analyses will be performed for pre-intervention variables (CRP, Hb, leukocyte, creatinine and pain) and if statistically significant differences between groups will be found, then ROC-analysis will be performed to find a cut-point value for variable in order to predict complicated AA. A ROC-curve, sensitivity and specificity will be presented as the results of analyses.

All patients in the antibiotic group who have not undergone appendectomy within the 10-year follow-up period, will undergo abdominal MRI (without radiation exposure) to assess the intact appendix to rule out any missed appendiceal neoplasms, regardless of the presence of symptoms. In the five-year follow of the study^4^, none of the patients in the antibiotic treatment group who later underwent surgery were found to have an appendiceal tumor. If a patient has a contraindication to MRI, (e.g., metal in the body), alternative imaging options will be discussed with the patient, and their use will be evaluated based on the clinical situation. Any abnormal imaging findings will be managed according to clinical practice guidelines. This enhances the ethical integrity of the study even though the likelihood of detecting appendiceal tumors is low.

**5. Cost analysis**

All related costs will be estimated based on the actual input terms of resource use and personnel in the 12-month follow-up period after randomization. All costs will be derived from the Finnish hospital cost or determined in co-operation with the hospital administration. Direct medical costs will be recorded in the case record forms. Indirect costs arising from losses in productivity will be assessed by means of the Health and Labor questionnaire and will be calculated by means of the friction cost method.

If the patient in the 10-year follow-up has symptoms, imaging and follow-up visits will be covered as part of the standard medical care. The imaging costs for asymptomatic patients will be covered by research grants.

**6. Follow-up**

The main results of the trial will be reported after one year follow-up of the patients. After that the patients will be interviewed also at 3, 5, and 10 years after treatment and the data is evaluated in every time-point.

At 7-year a post-hoc follow-up of the APPAC study^5^ was added to the study protocol to assess the patient quality of life (QOL) and satisfaction with the treatment by using a validated and standardized quality of life questionnaire (EQ-D-5L, validated in both Finnish and Swedish). The questionnaire includes five standardized questions about general quality of life, and in addition, the patients will be asked two additional questions: 1) Satisfaction with the treatment they received, and 2) if, they have chosen the randomized treatment at retrospect. The QOL assessment was also scheduled for the 10-year follow-up. The interviews were conducted by a phone interviews.

**References**

1. Mason, R.J., A. Moazzez, H. Sohn, and N. Katkhouda, **Meta-analysis of randomized trials comparing antibiotic therapy with appendectomy for acute uncomplicated (no abscess or phlegmon) appendicitis***.* Surg Infect (Larchmt), 2012. **13**(2): p. 74-84.
2. Salminen P, Tuominen R, Paajanen H, et al. Five-year Follow-up of Antibiotic Therapy for Uncomplicated Acute Appendicitis in the APPAC Randomized Clinical Trial. JAMA. Sep2 5 2018;320(12):1250-1265. doi:10.1001/jama.2018.13201
3. Sippola S, Haijanen J, Viinikainen L, et.al. Quality fo Life and Patient Satisfaction at 7-year Follow-up of Antibiotic Therapy vs Appendectomy for Uncomplicated Acute Appendicitis: A secondary Analysis of a Randomized Clinical Trial. JAMA Surg. Apr 1 2020;155(4):283-289. doi:10.1001/jamasurg.2019.6028

**Supplement 3. The APPAC II trial protocol** 27.10.2015

**Optimizing the antibiotic treatment of uncomplicated acute appendicitis: a prospective randomized multicenter study**

**Table of contents**

1. Background

1.1. The APPAC trial

1.2. The diagnosis and treatment of acute appendicitis

1.2.1. Uncomplicated and complicated acute appendicitis

1.2.2. Computed tomography (CT) in diagnosing acute appendicitis

1.2.3. Treatment of acute appendicitis

2. Aims of the study and study hypothesis

3. Combination of APPAC II and APPAC III studies in clinical practice

4. Combination of APPAC II and MAPPAC studies in clinical practice

5. Study design, patients and methods

5.1. Trial design

5.2. Participants

5.3. Registration and randomization

5.4. Interventions

5.5. Outcome parameters

5.6. Data collection and follow-up

6. Statistical methods

6.1. Statistical hypothesis

6.2. Sample size calculations

6.3. Interim analysis

6.4. Statistical analysis

7. Ethical considerations and study relevance

8. Study costs

9. Study schedule

10. Study hospitals and investigators

11. References

**1. Background**

Appendectomy has unquestionably been the standard treatment for acute appendicitis for over a century. More than 300.000 appendectomies are performed annually in the United States^1^. Although appendectomy is generally well tolerated, it is a major surgical intervention and can be associated with postoperative morbidity ^2, 3^.

Since the time Fitz described the relationship between the appendix and pelvic abscess and McBurney demonstrated reduced morbidity from pelvic infections attributable to appendectomy, it has been thought that acute appendicitis invariably progresses to perforation. This line of thinking underlies the belief that emergency appendectomy is required when a diagnosis of appendicitis is made ^4, 5^. Fitz and McBurney’s publications predated the availability of antibiotics by 40 years. In the absence of antibiotics, appendectomy saved lives by reducing the risk of uncontrolled pelvic infection when appendicitis was present.

Even though appendectomy has been the mainstay treatment for appendicitis, relatively soon after antibiotics were available, Coldrey reported treating 471 acute appendicitis patients with antibiotic therapy in 1959. Mortality was low (0.2 %) and recurrent appendicitis occurred only in 14.4 % of patients ^6^. More recently, the notion of treating appendicitis with antibiotics was tested in 3 randomized clinical trials (Table 1^7^) ^8-10^.Their results were summarized in a Cochrane analysis ^11^ and several meta-analyses. ^12-16^ Each of these trials had limitations and appendectomy has remained the standard approach for treating appendicitis.

- 1. **The APPAC trial**

In order to compare antibiotic therapy with appendectomy in the treatment of CT-scan confirmed uncomplicated acute appendicitis, we conducted the APPAC trial enrolling patients from November 2009 to June 2012. The APPAC trial ^7^ is a multicenter, randomized, open-label, non-inferiority trial conducted in Finland enrolling 530 patients 18 to 60 years of age with a CT scan confirmed uncomplicated acute appendicitis. Patients were randomly assigned to early appendectomy or antibiotic treatment with a follow-up of one year. Antibiotic therapy was intravenous ertapenem for three days followed by seven days of oral levofloxacin and metronidazole treatment; patients randomized to the operative treatment group underwent standard, open appendectomy.

The primary endpoint for surgical intervention was the successful completion of an appendectomy. The primary endpoint for antibiotic treated patients was discharge from the hospital without the need for surgery and no recurrent appendicitis during a follow-up of one-year.

A pre-specified non-inferiority margin of 24 percentage points for the difference between treatments was used. Secondary pre-specified endpoints included hospital stay, post-intervention pain, sick leave and overall morbidity.

There were 273 patients in the operative group and 257 in the antibiotic group. All but 1 patient in the surgery group underwent successful appendectomy resulting in a 100% (272/273; CI 98.9 – 100.0) success rate. In the antibiotic group, 70 patients (27.3 %: (CI 22.0 – 33.2) underwent surgical intervention within 1 year of initial presentation for appendicitis and 186 of 256 patients available for follow-up (72.7%; CI 66.8 – 78.0) did not require surgery. The intent-to-treat analysis yielded a success rate difference of 27.3 % (CI 22.0 – 33.2). Given our pre-specified non-inferiority margin of 24%, we were unable to demonstrate non-inferiority of antibiotic treatment relative to surgery. Of the patients randomized to antibiotic treatment, who subsequently underwent appendectomy, 58 (82.9%; CI 72.0 – 90.8) had uncomplicated appendicitis, 7 (10.0%; CI 4.1 – 19.5) had complicated appendicitis and 5 (7.1%; CI 2.4 – 15.9) operated for suspected recurrence did not have appendicitis. There were no intra-abdominal abscesses or other major complications associated with delayed appendectomy in this group.

Antibiotic treatment of patients with uncomplicated acute appendicitis was not shown to be non-inferior to appendectomy for uncomplicated appendicitis within the first year of observation following initial presentation of appendicitis. The pre-specified non-inferiority margin was established somewhat arbitrarily because little clinical information was available to make a better estimate. However, the majority (73%), of patients with uncomplicated acute appendicitis were successfully treated with antibiotics. None of the patients, who initially were treated with antibiotics that later had appendectomy, had major complications. These results suggest that CT-proven uncomplicated acute appendicitis is not a surgical emergency and antibiotic therapy is a safe first-line treatment of uncomplicated acute appendicitis. With the development of more precise diagnostic capabilities like CT and effective broad-spectrum antibiotics, appendectomy may be unnecessary for uncomplicated appendicitis, which occurs in the majority of acute appendicitis cases. Patients should be able to make an informed decision between antibiotic treatment and appendectomy and focus should also be on taking into account the patient-centric outcomes. Future studies should focus both on early identification of complicated acute appendicitis patients needing surgery and to prospectively evaluate the optimal use of antibiotic treatment in patients with uncomplicated acute appendicitis.

**1.2. The diagnosis and treatment of acute appendicitis**

Acute appendicitis is the most common cause of abdominal pain in emergency departments and appendectomy is the most common emergency abdominal surgery. The lifetime risk of acute appendicitis in males is 8.6% and 6.7% in females. ^17^ In Finland according to Stakes data there were 6 377 appendectomies (3242 in males, 3135 in females, median age 35 years) performed in 2010. The total number of days in hospital care was 16 111 days and the mean length of hospital stay was three days.

Although acute appendicitis is the most common reason for surgical emergency department visit, its diagnosis still remains challenging. The clinical diagnosis has previously been based on patient history, physical examination and laboratory findings as well as the clinical surgical diagnosis. Several scoring systems have been created to aid in the diagnosis of acute appendicitis^18-20^, but the accuracy of clinical diagnosis without preoperative imaging is about 76 – 80 % for combined patient groups of males and females^21, 22^.

As acute appendicitis has historically been thought to always progress to perforation requiring emergency appendectomy, high negative appendectomy rates even up to 40 % in some patient populations have been previously accepted as good surgical practice. For the last two decades, the use of dedicated imaging in acute abdomen in general and also in acute appendicitis has led to improved diagnostic accuracy.

**1.2.1. Uncomplicated and complicated acute appendicitis**

Based on large epidemiological studies, we now know that complicated (perforated) and uncomplicated (non-perforated) appendicitis have followed different epidemiological trends. These unassociated epidemiologic trends suggest different pathophysiology for the two form of appendicitis. The differential diagnosis is essential as patients with an uncomplicated acute appendicitis may not require surgical intervention and might experience even spontaneous resolution without perforation.^23^ The majority (approximately 80 %) of acute appendicitis cases are of uncomplicated nature.

Complicated acute appendicitis defined as a finding of a perforation, appendicolith, abscess or a suspicion of a tumor, requires emergency appendectomy with the exception of cases with abscess as they are often managed conservatively.

Appendicolith is a calcified fecal concretion in the appendix resulting in internal luminal obstruction and it is the most common form of complicated acute appendicitis. In the first randomized study by Vons et al.^10^ comparing operative treatment and antibiotic therapy using CT as a diagnostic inclusion criterion, the presence of an appendicolith in preoperative CT scan was the only factor that significantly increased the risk of complicated appendicitis and it was also the only factor associated with the failure of antibiotic therapy for acute appendicitis. Indeed, if Vons et al^10^ had excluded the patients with an appendicolith from their analysis, no significant difference in the incidence of post-intervention peritonitis between the treatment groups would have been noticed in their study.

**1.2.2. Computed tomography (CT) in diagnosing acute appendicitis**

CT imaging is the primary imaging modality and the golden standard in the diagnosis of acute appendicitis as it establishes the diagnosis with almost perfect diagnostic accuracy. The advantages of CT imaging are high accuracy, availability, ease of performance and interpretation, and that it is rarely affected by bowel gas, severe abdominal pain or extreme body habitus. The main disadvantage of CT is exposure to radiation.

The increased use of preoperative CT imaging has been evaluated thoroughly by evaluating its impact on the negative appendectomy rate reducing the number of unnecessary appendectomies. In 2010, a mandatory imaging guideline for suspected acute appendicitis was implemented in the Netherlands. After implementation the negative appendectomy rate dropped significantly from 23 % to 6 % (p<0.001) reducing the surgical complication rate from 20% to 14 % and resulting in average cost-per-patient decrease by 594€.^24^

The favorable diagnostic performance of CT imaging has encouraged optimization of the protocol to minimize exposure to radiation through the development of low-dose CT protocols. Low-dose protocols balance with as low as reasonably achievable-principle while maintaining diagnostic accuracy. However, low-dose protocols with intravenous contrast are still not implemented in routine clinical practice. These protocols require more advanced optimization and validation because of the wider need for contrast enhanced assessment. Kim et al^25^ showed that contrast enhanced low-dose CT (median radiation dose 116mmGy in dose-length product) was not inferior to standard-dose contrast enhanced CT (median radiation dose 521 mmGy), with negative appendectomy rates of 3.5% and 3.2% respectively and no statistical significance in appendiceal perforation rates or patients requiring additional imaging.

We have initiated a prospective observational study (OPTICAP trial, NCT02533869, Ethical committee of Turku University Hospital approval) in order to optimize a low-dose CT scan for both diagnosing acute appendicitis and to differentiate uncomplicated acute appendicitis from a complicated acute appendicitis. In this study we have performed phantom imaging with 15 different imaging protocols aiming to minimize radiation with optimal diagnostic accuracy. The phantom protocols were assessed by blinded evaluation of two gastrointestinal radiologists and the two best performing protocols were chosen for the clinical phase. The clinical evaluation included performing both of these imaging protocols for patients with suspected uncomplicated acute appendicitis evaluated by a senior digestive surgeon. All of the enrolled patients underwent laparoscopic appendectomy to evaluate the sensitivity and specificity of the imaging protocols. The most optimal imaging protocol will be selected for use in the APPAC II and III trials; the final results will be available in September 2016.

**1.2.3. Treatment of acute appendicitis**

For over a century appendectomy has been the standard treatment for all patients with acute appendicitis. However, the results of our APPAC trial have now shown that the majority (73%) of patients with uncomplicated acute appendicitis were successfully treated with antibiotics alone. We also showed that none of the patients treated initially with antibiotics and later undergoing appendectomy had major complications or increased morbidity defining antibiotic therapy as a safe first-line treatment. Patients with a complicated acute appendicitis require emergency appendectomy and early identification of these patients is of vital importance. Laparoscopic appendectomy has become the golden standard for appendectomy providing lower morbidity and faster recovery compared with open appendectomy. For patients with uncomplicated acute appendicitis, the time has come to evaluate abandoning routine appendectomy and evaluating the optimal use of antibiotic therapy.

**2. Aims of the study and study hypothesis**

The aim of study is to optimize the antibiotic therapy for uncomplicated acute appendicitis by evaluating the success of treatment in both study groups and by comparing intravenous antibiotic therapy followed by per oral antibiotics with per oral antibiotic monotherapy. The study hypothesis is that broad-spectrum intravenous antibiotics requiring additional hospital resources are not necessary for the treatment of uncomplicated acute appendicitis and that per oral monotherapy is non-inferior to the combination of intravenous and per oral antibiotic therapy. The secondary aim is to evaluate the results of our randomized APPAC trial in a prospective patient cohort by implementing antibiotic therapy as the first-line treatment for uncomplicated acute appendicitis in clinical practice.

**3. Combination of APPAC II and APPAC III studies in clinical practice**

APPAC II and APPAC III trials are separate studies regarding the applied study permissions (Fimea, Tukija, the Ethical committee of Turku University Hospital). In practice these two studies will be performed in close conjunction with each other as the enrolled patient population is identical in both studies and the study chosen for enrollment will be based mainly on the time of day (based on study design APPAC III enrollment is only possible between 8 a.m. and 2 p.m.) and secondly on patient preference (if the patient is unwilling to participate in APPAC III, they will be informed and invited to participate in APPAC II trial). After 2 p.m. until 8 a.m. all of the eligible patients will be invited to participate in APPAC II trial.

**4. Combination of APPAC II and MAPPAC studies**

The MAPPAC trial assesses the microbiological etiology of appendicitis and the impact of antibiotic therapy on gut microbiota. MAPPAC trial will be enrolling in conjunction with the APPAC II and III trials. APPAC II and MAPPAC trials are separate studies regarding the applied study permissions (Fimea, Tukija, the Ethical Committee of Turku University Hospital). Patients recruited for the APPAC II trial will asked to sign an informed consent form allowing for the use of their data and collection of microbiological samples for the MAPPAC study and vice versa MAPPAC trial patients will be informed that MAPPAC study data will be used in conjunction with APPAC II trial.

**5. Study design, patients and methods**

**5.1. Trial design**

The APPAC II trial has been designed as a prospective randomized open-label, non-inferiority multicenter trial to compare intravenous antibiotic therapy followed by per oral antibiotics with per oral antibiotic monotherapy in the treatment of uncomplicated acute appendicitis.

**5.2. Participants**

Patients presenting with suspected acute appendicitis will be enrolled from eight participating Finnish hospitals; four university hospitals (TYKS, OYS, TAYS, KYS) and four central hospitals (Jyväskylä, Mikkeli, Hämeenlinna, Vaasa). All adult patients (aged 18 – 60 years) admitted to the emergency department with a clinical suspicion of uncomplicated acute appendicitis will undergo a low-dose CT scan optimized for the diagnosis of acute appendicitis (OPTICAP trial, please see chapter 1.2.2.). Clinical history, physical investigation, VAS pain scores (visual analogue scale) and laboratory tests will be recorded for all of the evaluated patients in a prospective online database (BCB Medical APPAC-database developed by our study group). An informed consent will be obtained from all of the patients.

Inclusion and exclusion criteria for both APPAC II and III trials are identical.

Inclusion criteria: 1) Signed informed consent, 2) Age 18 – 60 years, 3) CT scan confirmed diagnosis of uncomplicated acute appendicitis.

Exclusion criteria: 1) Age <18 or > 60 years, 2) Pregnancy or lactating, 3) Allergy to contrast media or iodine, 4) Allergy or contraindication to antibiotic therapy 5) Renal insufficiency, 6) Metformine medication, 7) Severe systemic illness (for example malignancy, medical condition requiring immunosuppressant medications), 8) Complicated acute appendicitis in a CT scan (appendicolith, perforation, abscess, suspicion of a tumor), 9) Inability to co-operate and give informed consent.

Contraindications for the use of antibiotics include either allergy to the antibiotic regimen or auxiliary substance or interaction with other medications. In the case of quinolones, epilepsy and previously diagnosed tendinitis or tendon rupture related to quinolone treatment are contraindications. With moxifloxacin, additional contraindications are liver failure, heart condition (for example prolonged QT-time) or electrolyte imbalance. Other overall contraindications to antibiotic treatment in general include pregnancy, lactation, and the age under 18 years; do not apply as these patients will not be evaluated for enrollment in the study based on exclusion criteria.

According to the study protocol all patients admitted to the emergency room with suspected acute appendicitis will undergo CT imaging as CT has become the golden standard imaging in diagnosing acute appendicitis. Based on our OPTICAP-trial, the CT scan protocol used for acute appendicitis will be optimized for radiation exposure. If complicated acute appendicitis is diagnosed on CT, patients will undergo a laparoscopic appendectomy within eight hours (the patients will be classified as “requiring surgery within 0- 8 hours” in an acute care surgery criteria used in the operating theatre). In order to collect all acute appendicitis patients both to prevent any bias and to enable a thorough conception of acute appendicitis as an emergency abdominal condition, all patients with suspected acute appendicitis undergoing a CT will be thoroughly recorded, the patients will be informed about data collection and an informed consent will be obtained. Additionally, an extra serum sample will be collected for later immunological analyses regarding patients presenting with complicated acute appendicitis (MAPPAC trial) and patients enrolled in APPAC II trial.

**5.3. Registration and randomization**

Patients will be randomized with a 1:1 equal allocation ratio to i.v. + p.o. or p.o. antibiotics group. The randomization procedure will be performed by a safety statistician of the trial. Randomization will be made by center using random permuted blocks. After evaluating the patient eligible for enrollment in APPAC II trial, the sealed and opaque randomization envelope will be opened by the surgeon on call in each participating hospital.

**5.4. Interventions**

In the APPAC II trial, the treatment arms will be intravenous (i.v.) + per oral (p.o.) vs. p.o. antibiotics and the duration of the antibiotic therapy in both treatment groups will be seven days. For patients randomized to i.v. + p.o. group, i.v. ertapenem sodium 1 g will be administered for two days with the first dose given in the emergency room. The i.v. ertapenem will be followed by p.o. levofloxacin 500 mg x 1 and metronidazole 500 mg x 3 for five days. For patients randomized to po group, p.o. moxifloxacin 400 mg will be administered for seven days with the first dose given in the emergency room. The minimum follow-up at the hospital will be 20 – 24 hours.

If the patient is suspected of not responding to the antibiotic therapy during the primary hospitalization, the following outcome parameters (VAS/changes in VAS, leukocyte count, CRP, temperature, status findings) will be registered in the database. To ensure patient safety in cases of suspected progression of the acute appendicitis, the patient will be operated on based on the surgeon’s decision. The operative finding and the histopathology of the appendix will be recorded in the database.

After the initial hospitalization recurrent acute appendicitis will be diagnosed on a clinical basis and a patient with a suspected recurrence will undergo laparoscopic appendectomy and the recurrent acute appendicitis will be verified by histopathological examination of the removed appendix. In cases of patients undergoing appendectomy for treatment failure, we will inform the patients, that further specialized histopathological analysis may be performed in addition to standard histopathological examination.

An extra serum sample will be obtained for future immunological analyses and all enrolled APPAC II patients are informed of collecting this extra serum sample and about performing immunological and possible other analysis using this acquired extra serum sample; this information is stated in the informed consent.

**5.5. Outcome parameters**

The primary endpoint is to estimate the success of the randomized treatment (treatment efficacy). Treatment success is defined as the resolution of acute appendicitis with antibiotic treatment resulting in discharge from the hospital without the need for surgical intervention and no recurrent appendicitis during a follow-up of one-year. In addition, the success of treatment will be compared between study groups. Secondary endpoints include post-intervention complications (Clavien-Dindo classification), late recurrence (after one-year) of acute appendicitis after antibiotic treatment, duration of hospital stay, VAS scores, quality of life (QOL, 15D), sick leave and treatment costs.

The MAPPAC study feces samples regarding APPAC II trial patients will be stored according to regulations. These samples will be used only for MAPPAC and APPAC II trials. All participating patients in each trial will be informed about combining the MAPPAC and APPAC II data.

**5.6. Data collection and follow-up**

After signed informed consent, all of the patients evaluated for acute appendicitis and study enrollment are registered to an online database at each participating institution. The researchers together with BCB Medical have created the online database, where all patients evaluated for enrollment in the study will be recorded. To ensure thorough data collection and to be able to evaluate selection bias, all of the patients presenting with acute appendicitis at the research hospitals will be recorded in the database. The information recorded from the patients who are not participating in APPAC II or APPAC III studies is used only by the regulations of register based studies. These patients not included in either of the APPAC trials will be informed of this data collection according to the guidelines of the Finnish health Ministry (STM) and their informed consent will be obtained. The data collection will be sent online to the database and Turku University Hospital as the main research center will be in charge of the common database with full access to the data. The researchers need the full access to the data in order to be able to correct possible false data entries, to enter possible missing data and to be able to keep up with the number of enrolled patients. The online database will not be used for other purposes during the trial and all of the visits to the database will be recorded in the database log.

The follow-up for patients in APPAC II trial will include a phone interview at one week, two months and at one, three, five and ten years.

**6. Statistical methods**

**6.1 Statistical hypothesis**

The primary objective of the study is to demonstrate that p.o. antibiotics are adequate treatment for uncomplicated acute appendicitis. The primary outcome is success of treatment and it will be evaluated in two stages using following statistical hypotheses:

1. H_0_: p_1_ ≤ 65 and p_2_ ≤ 65

H_1_: p_1_ > 65 and p_2_ > 65

1. H_0_: p_1_ - p_2_ > 6

H_1_: p_1_ - p_2_ ≤ 6

where p_1_ is success of treatment proportion of i.v. + p.o. group and p_2_ for p.o. group and p_1_ - p_2_ is difference between groups ((i.v. + p.o.) – po).

**6.2. Sample size calculations**

Sample size calculations were based on non-inferiority test for binomial proportion. Sample size was calculated from an estimated success rate of 73% for i.v. + p.o. antibiotic group during the 1 year follow-up^7^. The hypothetical difference between groups ((i.v. + p.o.) – p.o.) was set to zero and non-inferiority margin was set to 6 percentage points. We estimated that total of 469 patients would yield a power of 0.9 (1-β) to establish whether p.o. antibiotic therapy was non-inferior to i.v. + p.o. using a one-sided significance level (α) of 0.05. With an estimated dropout rate of 15% total of 552 patients, 276 patients per group will be enrolled in the study. Targeted minimum sample size per study hospital will be 20 patients. Sample size calculations were performed using Power procedure in SAS System for Windows, Version 9.4 (SAS Institute Inc., Cary, NC).

**6.3. Interim analyses**

When 250 patients are enrolled to the study and discharged from the hospital, or if the investigators think it is necessary, the point estimate of the success rate at discharge will be calculated by study statistician and evaluated in each group. If the proportion is below 70% in at least one of the groups, the study will be terminated. The whole study group will be informed of the group proportions and whether the study is allowed to continue or will be terminated. No statistical tests will be conducted in interim analysis and therefore no corrections to the p-values are needed in the final analyses of study.

**6.4. Statistical analyses**

Categorical variables of the study will be characterized by treatment using frequencies and percentages and for continuous variables means and standard deviations or medians with range and 25^th^ and 75^th^ percentiles will be used. The point estimate with 95% confidence interval (CI) for success of treatment will be calculated for both groups and if lower limit of 95% CI ≥ 65% then treatment is good enough. Non-inferiority of p.o. antibiotics vs. i.v. + p.o. antibiotics will be evaluated using a two-sided 90% CI of proportion difference between groups ((i.v. + p.o.) – p.o. antibiotics) and one-sided Wald test for non-inferiority with an α level of 0.05. Non-inferiority margin for difference is 6 percentage points. The secondary outcomes will be analyzed using chi squared test, independent samples t-test or Mann-Whitney U-test. The assumptions of tests will be checked for justification of the analyses. For the secondary outcomes two-sided p-values will be used. The study site differences will be evaluated in statistical models and if major differences are detected more complicated statistical models will be used in the analyses of primary and secondary outcomes. P-values less than 0.05 will be considered statistically significant. The analyses will be based on the intention-to-treat (ITT) principle (all randomized excluding possible erroneously randomized patients with CT diagnosis of complicated appendicitis). For the primary end-point, in cases of patients lost to follow-up, missing data will be gathered from hospital registries, if possible, but for secondary outcomes, the subjects with missing data will automatically be excluded from the analyses of the variables in concern. Statistical analyses will be performed using SAS System for Windows, Version 9.4 or later (SAS Institute Inc., Cary, NC).

**7. Ethical considerations and study relevance**

Both APPAC II and APPAC III study protocols are based on the results of our randomized APPAC trial comparing antibiotic therapy with appendectomy in the treatment of uncomplicated acute appendicitis. Based on the results of our APPAC trial, we now know that the majority (73%) of patients with uncomplicated acute appendicitis can be safely treated by antibiotics alone and that none of the patients, who initially were treated with antibiotics that later had appendectomy, had major complications. These results suggest that CT-proven uncomplicated acute appendicitis is not a surgical emergency and antibiotic therapy is a safe first-line treatment of acute uncomplicated appendicitis. The APPAC study results are based on the accurate diagnosis of acute appendicitis and CT imaging has become the golden standard in diagnosing acute appendicitis. Prior to initiation of the APPAC II and III trials, we are aiming to minimize the radiation exposure by optimizing a low-dose CT protocol combining high sensitivity and specificity with markedly reduced radiation exposure (the OPTICAP trial). The APPAC II trial will evaluate the treatment of uncomplicated acute appendicitis with two different antibiotic therapies aiming to optimize the antibiotic treatment by shortening the duration of the treatment, taking into account the antibiotic resistance problem by evaluating less broad-spectrum antibiotics and minimizing the required hospital stay.

The relevance of our previous APPAC trial has been substantial in initiating worldwide discussion and evaluation of the optimal treatment for uncomplicated acute appendicitis as the time has come to abandon routine appendectomy for uncomplicated acute appendicitis. The changes in the treatment paradigm for CT-proven uncomplicated acute appendicitis will naturally require further prospective studies, but avoiding unnecessary appendectomies will result in major cost savings and markedly decreased operative morbidity. APPAC II trial results will further enhance the thorough evaluation of the use of antibiotic therapy in uncomplicated acute appendicitis as by optimizing the antibiotic treatment will result in further cost savings and better utilization of hospital resources. As we now have the results of our initial APPAC trial, the international study focus on acute appendicitis will be the evaluation of the non-operative management and its optimization. Based on our APPAC trial, we are in the frontline of this research even from an international point of view and both APPAC II/III study hypothesis are the key questions in this research field.

Acute appendicitis is one of the most common surgical emergencies and appendectomy is the most common surgical emergency operation with approximately 300.000 annual procedures in the US and 6500 appendectomies in Finland. The results of both the completed and future APPAC trials are very likely to have a profound impact on the treatment paradigm of uncomplicated acute appendicitis by avoiding unnecessary surgeries and the related morbidity resulting in major cost savings.

**8. Study costs**

Based on our APPAC trial results, antibiotic therapy is a safe first-line treatment for uncomplicated acute appendicitis. The diagnosis of acute appendicitis with a CT can be achieved with almost perfect diagnostic accuracy and CT imaging is now considered standard in diagnosing acute appendicitis. The APPAC II trial does not deviate from the standard care for acute appendicitis and thus there are no extra costs regarding the study interventions.

**9. Study schedule**

APPAC II/III trials require an optimal low-dose CT protocol for the diagnosis of acute uncomplicated appendicitis. The aim of the already initiated OPTICAP trial is to optimize the low-dose CT scan and the phantom imaging protocols have already been performed and analyzed during September 2015. The clinical phase of the OPTICAP trial started in October 2015 after the acceptance notification of OPTICAP amendment for the 20.10.2015 Ethical committee meeting. The clinical phase was initiated in November 2015; first 40 patients were enrolled by the end of April. The last 20 patients were enrolled in August, the data is being analyzed and the results will be available in September 2016. In October 2016 we will have an optimized low-dose CT protocol to be used in APPAC II/III trials.

The Finnish Society for Digestive Surgeons has chosen our APPAC II/III trials for creating an online research database for the society members to use as a basis for the database had to be built based on an actual trial. The costs for building such a database are 20.000€ and the costs are covered by the society. We have provided the clinical and scientific expertise for building this database, this work started already in November 2014 and the database will be used for APPAC II/III studies. The database is now finished and the programming by BCB Medical will be finished by the end of September 2016. The database will be available for use by the initiation of APPAC II trial in November 2016.

APPAC II trial protocol announcement has been already sent to the TUKIJA national committee for medical trials and their decision was to transfer the ethics committee evaluation to the local ethics committee. The APPAC II trial has been reported to and accepted by Fimea and the Eudra-CT codes are (2015-003633-10). The APPAC III trial is currently undergoing hospital pharmacy evaluations regarding the manufacturing of the trial medications. After the manufacturing plan is finished, APPAC III trial will be submitted to Fimea for final approval during the fall 2016.

Both APPAC II and III trials enroll patients from the same patient population, please see chapter 3. APPAC II trial enrollment is evaluated to last for approximately two years (until December 2018) and the primary endpoint will be analyzed at one-year follow-up at the end of 2019. APPAC III trial enrollment is scheduled to be completed by March 2018 and the primary endpoint at ten-day follow-up will be analyzed in April 2018. For both trials, the follow-up will extend to ten years.

**10. Study hospitals and investigators**

APPAC II trial will be a national multicenter study and Turku University Hospital will be the main research center and the primary investigator will be Paulina Salminen.

Study will be conducted at all four university hospitals (Turku, Tampere, Oulu and Kuopio) and four central hospitals (Jyväskylä, Mikkeli, Hämeenlinna, Vaasa).

The investigators at each research hospital: 1) Turku (Paulina Salminen, MD, PhD, Juha Grönroos, MD, PhD, Johanna Virtanen MD, PhD, Suvi **Sippola,** MD, PhD student, Harri Marttila, MD, PhD,), 2) Tampere (Pia Nordström, MD, PhD, Johanna Laukkarinen MD, PhD, Irina Rinta-Kiikka, MD, PhD), 3) Oulu (Tero Rautio MD, PhD, Sanna Meriläinen MD, PhD), 4) Kuopio (Hannu Paajanen MD, PhD, Tuomo Rantanen MD, PHD), 5) Jyväskylä (Markku Aarnio MD, PhD, Anne Mattila, MD), 6) Mikkeli (Imre Ilves, MD, Hannu Paajanen MD, PhD), 7) Lahti (Jyrki Kössi, MD, PhD, Juhani Sand, MD, PhD), 8) Rovaniemi (Jukka Rintala, MD, PhD), 9) Pori (Jussi Haijanen, MD, PhD student, Eeva-Liisa Sävelä, MD) and 10) Seinäjoki (Tarja Pinta, MD, PhD, Tomi Sippola, MD).

In addition, the study statistician is Saija Hurme, MSc (University of Turku) and the study health economics specialist is professor Petri Böckerman (University of Turku).

**11. References**

1.Livingston EH, Fomby TB, Woodward WA, Haley RW. Epidemiological similarities between appendicitis and diverticulitis suggesting a common underlying pathogenesis. *Archives of surgery*. 2011 Mar;146(3):308-14.

2.Leung TT, Dixon E, Gill M, et al. Bowel obstruction following appendectomy: what is the true incidence? *Ann Surg*. 2009 Jul;250(1):51-3.

3.Margenthaler JA, Longo WE, Virgo KS, et al. Risk factors for adverse outcomes after the surgical treatment of appendicitis in adults. *Ann Surg*. 2003 Jul;238(1):59-66.

4.McBurney C. Experience with early operative interference in cases of disease of the vermiform appendix. *N Y Med J*. 1889;50:676-84.

5.Fitz R. Perforating inflammation of the vermiform appendix. *Am J Med Sci*. 1886;92:321-46.

6.Coldrey E. Treatment of Acute Appendicitis. *Br Med J*. 1956 Dec 22;2(5007):1458-61.

7.Salminen P, Paajanen H, Rautio T, et al. Antibiotic Therapy vs Appendectomy for Treatment of Uncomplicated Acute Appendicitis: The APPAC Randomized Clinical Trial. *Jama*. 2015 Jun 16;313(23):2340-8.

8.Hansson J, Korner U, Khorram-Manesh A, Solberg A, Lundholm K. Randomized clinical trial of antibiotic therapy versus appendicectomy as primary treatment of acute appendicitis in unselected patients. *Br J Surg*. 2009 May;96(5):473-81.

9.Styrud J, Eriksson S, Nilsson I, et al. Appendectomy versus antibiotic treatment in acute appendicitis. a prospective multicenter randomized controlled trial. *World J Surg*. 2006 Jun;30(6):1033-7.

10.Vons C, Barry C, Maitre S, et al. Amoxicillin plus clavulanic acid versus appendicectomy for treatment of acute uncomplicated appendicitis: an open-label, non-inferiority, randomised controlled trial. *Lancet*. 2011 May 7;377(9777):1573-9.

11.Wilms IM, de Hoog DE, de Visser DC, Janzing HM. Appendectomy versus antibiotic treatment for acute appendicitis. *Cochrane Database Syst Rev*. 2011(11):CD008359.

12.Ansaloni L, Catena F, Coccolini F, et al. Surgery versus conservative antibiotic treatment in acute appendicitis: a systematic review and meta-analysis of randomized controlled trials. *Dig Surg*. 2011;28(3):210-21.

13.Liu K, Fogg L. Use of antibiotics alone for treatment of uncomplicated acute appendicitis: a systematic review and meta-analysis. *Surgery*. 2011 Oct;150(4):673-83.

14.Mason RJ, Moazzez A, Sohn H, Katkhouda N. Meta-analysis of randomized trials comparing antibiotic therapy with appendectomy for acute uncomplicated (no abscess or phlegmon) appendicitis. *Surg Infect (Larchmt)*. 2012 Apr;13(2):74-84.

15.Varadhan KK, Humes DJ, Neal KR, Lobo DN. Antibiotic therapy versus appendectomy for acute appendicitis: a meta-analysis. *World J Surg*. 2010 Feb;34(2):199-209.

16.Varadhan KK, Neal KR, Lobo DN. Safety and efficacy of antibiotics compared with appendicectomy for treatment of uncomplicated acute appendicitis: meta-analysis of randomised controlled trials. *BMJ*. 2012;344:e2156.

17.Addiss DG, Shaffer N, Fowler BS, Tauxe RV. The epidemiology of appendicitis and appendectomy in the United States. *Am J Epidemiol*. 1990 Nov;132(5):910-25.

18.Alvarado A. A practical score for the early diagnosis of acute appendicitis. *Ann Emerg Med*. 1986 May;15(5):557-64.

19.Andersson M, Andersson RE. The appendicitis inflammatory response score: a tool for the diagnosis of acute appendicitis that outperforms the Alvarado score. *World J Surg*. 2008 Aug;32(8):1843-9.

20.Sammalkorpi HE, Mentula P, Leppaniemi A. A new adult appendicitis score improves diagnostic accuracy of acute appendicitis--a prospective study. *BMC Gastroenterol*. 2014;14:114.

21.Berry J, Jr., Malt RA. Appendicitis near its centenary. *Ann Surg*. 1984 Nov;200(5):567-75.

22.Korner H, Sondenaa K, Soreide JA, et al. Incidence of acute nonperforated and perforated appendicitis: age-specific and sex-specific analysis. *World J Surg*. 1997 Mar-Apr;21(3):313-7.

23.Livingston EH, Woodward WA, Sarosi GA, Haley RW. Disconnect between incidence of nonperforated and perforated appendicitis: implications for pathophysiology and management. *Ann Surg*. 2007 Jun;245(6):886-92.

24.Lahaye MJ, Lambregts DM, Mutsaers E, et al. Mandatory imaging cuts costs and reduces the rate of unnecessary surgeries in the diagnostic work-up of patients suspected of having appendicitis. *European radiology*. 2015 Jan 16.

25.Kim K, Kim YH, Kim SY, et al. Low-dose abdominal CT for evaluating suspected appendicitis. *N Engl J Med*. 2012 Apr 26;366(17):1596-605.

**Supplement 4. APPAC II trial: Statistical analysis plan**

Jussi Haijanen, M.D, Suvi Sippola, M.D., Juha Grönroos, M.D., Ph.D., Saija Hurme, M.Sc., Eliisa Löyttyniemi, M.Sc., and Paulina Salminen, M.D., Ph.D., on behalf of the APPAC II study group

Version number 0.1

Responsibilities:

Approved by Saija Hurme 27.10.2015 / amendment 2.4.2020

Issued by and analyses will be conducted By Eliisa Löyttyniemi.

SAS-programming will be done by Teemu Kemppainen.

1. **Introduction**

APPAC II trial is a multicentre, open-label, non-inferiority randomized controlled trial comparing per oral (p.o.) antibiotic monotherapy with intravenous (i.v.) antibiotic therapy followed by p.o. antibiotics in the treatment of CT-scan confirmed uncomplicated acute appendicitis. Primary endpoint is the success of the randomized treatment, defined as resolution of acute appendicitis resulting in discharge from the hospital without the need for surgical intervention and no recurrent appendicitis during one-year follow-up. Secondary endpoints include post-intervention complications (possible postoperative complications classified using primarily the Clavien-Dindo classification^1^), late recurrence (after one year) of acute appendicitis after antibiotic treatment, duration of hospital stay, VAS scores, quality of life (QOL, using for example 5D or 15D validated QOL questionnaire), length of sick leave and treatment costs.

1. **Sample size calculation**

The primary objective of the study is to demonstrate that p.o. antibiotic therapy is non-inferior compared to a combination of i.v. and p.o. antibiotic therapy, i.e. the trial is designed as a non-inferiority study and sample size calculations were based on non-inferiority test for binomial proportion. Sample size was calculated from an estimated success rate of 73% for i.v. + p.o. antibiotic group during the one year follow-up based on the results of our previous APPAC trial^2^. The hypothetical difference between the two groups ((i.v. + p.o.) vs (p.o.)) was set to zero and non-inferiority margin was set to 6 percentage points. We estimated that a total of 469 patients would yield a power of 0.9 (1-β) to establish whether p.o. antibiotic therapy was non-inferior to i.v. + p.o. using a one-sided significance level (α) of 0.05. With an estimated dropout rate of 15% total of 552 patients, 276 patients per group will be enrolled in the study. Targeted minimum sample size per study hospital will be 20 patients.

1. **Interim analysis**

When 250 patients have been enrolled to the study and discharged from the hospital, or even at an earlier stage if the investigators think it is necessary, the point estimate of the success rate at discharge will be calculated by study statistician and evaluated in each group to ensure patient safety. If the proportion is below 70% in at least one of the groups, the study will be terminated. No statistical tests will be conducted in interim analysis and therefore no corrections to the p-values are needed in the final analyses of the study.

1. **Statistical analysis**

Categorical variables of the study will be characterized using frequencies and percentages. For continuous variables means and standard deviations, or medians with range and 25^th^ and 75^th^ percentiles will be used. The study site differences will be evaluated in statistical models and if major differences are detected, more complicated statistical models will be used in the analyses of primary and secondary outcomes. P-values less than 0.05 will be considered statistically significant. The analyses will be based on the intention-to-treat (ITT) principle (all randomized excluding possible erroneously randomized patients with a primary CT diagnosis of complicated appendicitis). Statistical analyses will be performed using SAS System for Windows, Version 9.4 or later (SAS Institute Inc., Cary, NC).

- 1. **Primary outcome**

The primary objective of the study is to demonstrate that p.o. antibiotic therapy is as efficient and safe as i.v. + p.o. antibiotics for CT-scan confirmed uncomplicated acute appendicitis. The primary outcome will be evaluated in two stages using the following statistical hypotheses:

1. H_0_: p_1_ ≤ 65 and p_2_ ≤ 65

H_1_: p_1_ > 65 and p_2_ > 65

1. H_0_: p_1_ - p_2_ > 6

H_1_: p_1_ - p_2_ ≤ 6

where p_1_ is success of treatment proportion in i.v. + p.o. group and p_2_ for p.o. group and p_1_ - p_2_ is the difference between the groups ((i.v. + p.o.) – po).

The point estimate with 95% confidence interval (CI) for success of treatment will be calculated for both groups and if lower limit of 95% CI ≥ 65%, then treatment is good enough. Non-inferiority of p.o. antibiotics vs. i.v. + p.o. antibiotics will be evaluated using a two-sided 90% CI of proportion difference between groups and one-sided Wald test for non-inferiority with an α level of 0.05. Non-inferiority margin for difference is 6 percentage points. In cases of patients lost to follow-up, for the primary end-point of treatment success and the secondary endpoints of late recurrence, morbidity, and mortality, missing data will be retrieved from hospital registries.

Regarding the primary outcome, multiple prognostic factors potentially effecting the primary outcome will also be analyzed; for example patient age, gender, body mass index, laboratory test values, fever, VAS score, symptoms, and symptom duration prior to hospitalization as well as imaging features such as appendiceal diameter, minor fluid or edema around the appendix.

- 1. **Secondary outcomes**

The secondary outcomes will be analyzed using chi-squared test, independent samples t-test or Mann-Whitney U-test. The assumptions of tests will be checked for justification of the analyses. For the secondary outcomes two-sided p-values will be used. The subjects with missing data will automatically be excluded from the analyses of the variables in concern.

The secondary outcomes will be evaluated primarily at the predefined follow-up time points, excluding the comprehensive QOL life assessment, which will mainly be carried out at the 3 year time point, considering the current understanding that most of the recurrences after antibiotic treatment will occur during the first 2 years after initial treatment.^3^ In a small subset of patients, earlier QOL data will be retrieved to assess the potential differences.

- 1. **Subgroup analyses**

Additional analyses will be performed for overall morbidity using intention-to-treat principle.

1. **Follow-up**

The primary outcome results of the trial will be assessed after one-year-follow-up of the patients. The patients will be assessed for the secondary outcomes also at 3, 5 and 10 years after treatment and the data is evaluated in every time-point. If the primary endpoint analysis at 1 year shows non-inferiority of the p.o. antibiotic therapy, this primary endpoint will also be assessed at further follow-up time points.

1. Clavien PA, Barkun J, de Oliveira ML, et al. The Clavien-Dindo classification of surgical complications: five-year experience. *Ann Surg.* 2009;250(2):187-196.

2. Salminen P, Paajanen H, Rautio T, et al. Antibiotic Therapy vs Appendectomy for Treatment of Uncomplicated Acute Appendicitis: The APPAC Randomized Clinical Trial. *JAMA.* 2015;313(23):2340-2348.

3. Salminen P, Tuominen R, Paajanen H, et al. Five-Year Follow-up of Antibiotic Therapy for Uncomplicated Acute Appendicitis in the APPAC Randomized Clinical Trial. *JAMA.* 2018;320(12):1259-1265.
